# De novo biosynthesis of bioactive isoflavonoids by engineered yeast cell factories

**DOI:** 10.1038/s41467-021-26361-1

**Published:** 2021-10-19

**Authors:** Quanli Liu, Yi Liu, Gang Li, Otto Savolainen, Yun Chen, Jens Nielsen

**Affiliations:** 1 Department of Biology and Biological Engineering, Chalmers University of Technology, Kemivägen 10, SE-412 96 Gothenburg, Sweden; 2 Novo Nordisk Foundation Center for Biosustainability, Chalmers University of Technology, SE-412 96 Gothenburg, Sweden; 3 Chalmers Mass Spectrometry Infrastructure, Chalmers University of Technology, Kemivägen 10, SE-412 96 Gothenburg, Sweden; 4 Institute of Public Health and Clinical Nutrition, University of Eastern Finland, FI-70211 Kuopio, Finland; 5 Novo Nordisk Foundation Center for Biosustainability, Technical University of Denmark, 2800 Kongens Lyngby, Denmark; 6 BioInnovation Institute, Ole Maaløes vej 3, 2200 Copenhagen N, Denmark

**Keywords:** Metabolic engineering, Synthetic biology, Applied microbiology, Biocatalysis

## Abstract

Isoflavonoids comprise a class of plant natural products with great nutraceutical, pharmaceutical and agricultural significance. Their low abundance in nature and structural complexity however hampers access to these phytochemicals through traditional crop-based manufacturing or chemical synthesis. Microbial bioproduction therefore represents an attractive alternative. Here, we engineer the metabolism of *Saccharomyces cerevisiae* to become a platform for efficient production of daidzein, a core chemical scaffold for isoflavonoid biosynthesis, and demonstrate its application towards producing bioactive glucosides from glucose, following the screening-reconstruction-application engineering framework. First, we rebuild daidzein biosynthesis in yeast and its production is then improved by 94-fold through screening biosynthetic enzymes, identifying rate-limiting steps, implementing dynamic control, engineering substrate trafficking and fine-tuning competing metabolic processes. The optimized strain produces up to 85.4 mg L^−1^ of daidzein and introducing plant glycosyltransferases in this strain results in production of bioactive puerarin (72.8 mg L^−1^) and daidzin (73.2 mg L^−1^). Our work provides a promising step towards developing synthetic yeast cell factories for de novo biosynthesis of value-added isoflavonoids and the multi-phased framework may be extended to engineer pathways of complex natural products in other microbial hosts.

## Abstract (web summary)

Isoflavonoids are a class of industrially important plant natural products, but their low abundance and structural complexity limits their availability. Here, the authors engineer *Saccharomyces cerevisiae* metabolism to become a platform for efficient production of daidzein which is core chemical scaffold for isoflavonoid biosynthesis, and show its application for production of bioactive glucosides from glucose.

## Introduction

Isoflavonoids constitute a diverse family of natural products that are primarily synthesized by leguminous plants^1^. In addition to playing significant ecological functions^2^, isoflavonoids exhibit various human health-promoting properties, such as antioxidant activity, cardioprotective activity, osteoporosis reduction, and cancer prevention, all of which have resulted in studies on exploiting these molecules as agents both in the pharmaceutical and nutraceutical industry^3,4^. The current production of isoflavonoids relies on direct plant extraction. However, the low phytochemical abundance, significant investment of time, energy, and capital, and huge requirement for potentially toxic solvents have excluded this approach from being used as it is neither economical nor environmental-friendly^5,6^. Moreover, the cultivation of legumes is geographically uneven and the amounts of isoflavonoids vary greatly from cultivars and climatic conditions^7^. All these facts introduce further risk and instability in the supply of these chemicals by means of plant extraction. Developing alternative sources of isoflavonoids is therefore a prominent challenge to be addressed, prior to being able to feasibly produce these chemicals at scale using standardized industrial processes.

With the rapid advance in metabolic engineering and synthetic biology over the last few decades, microbe-based bioproduction has become increasingly pursued as an alternative to traditional chemical production techniques^8,9^. By re-engineering the cellular metabolism of fast-growing microorganisms, such as *Escherichia coli* and *Saccharomyces cerevisiae*, artificial cell platforms have been successfully constructed to produce high levels of chemicals ranging from biofuels to proteins^10^. In addition, grafting and optimizing plant biosynthetic pathways in microbial hosts is becoming a compelling route to supply plant natural products, as demonstrated by substantial biosynthesis of high-value-added alkaloids, stilbenes, and flavonoids, and terpenoids from simple sugar^11–15^. Based on this growing body of work, we speculated that microbial cell factories may also offer the potential for the production of commercially viable isoflavonoid as well.

Structurally, isoflavonoids contain the common C6-C3-C6 flavonoid skeleton and are characterized by having the B-ring connected at C3 rather than C2 position of the C-ring, compared to other flavonoid subclasses^3^ (Supplementary Fig. 1). The isoflavones genistein (GEIN) and daidzein (DEIN) constitute two basic scaffolds from which over thousands of isoflavonoids are derived as a result of diverse structural modifications, including hydroxylation, methylation, glycosylation, and molecular rearrangements^2–4^. Reconstruction of the isoflavone pathway for biosynthesis of these molecules, therefore, represents the entry point to microbial production of a large variety of different biologically active isoflavonoids. Previously, heterologous biosynthesis of GEIN and DEIN was demonstrated by introducing plant enzymes alongside feeding precursors, such as L-tyrosine, naringenin, or liquiritigenin, in both *E. coli* and *S. cerevisiae*^16–21^. Moreover, the expression of specific glucosyltransferase in *E. coli* also enabled the bioconversion of GEIN and DEIN to corresponding glucosides genistin (GIN) and daidzin (DIN)^22,23^, the primary form of stored isoflavones in leguminous plants^3^. While the reported low titers necessitate further improvement to support industrial-scale production, there have been rare efforts to engineer and optimize de novo microbial biosynthesis of isoflavones.

**Fig. 1:**
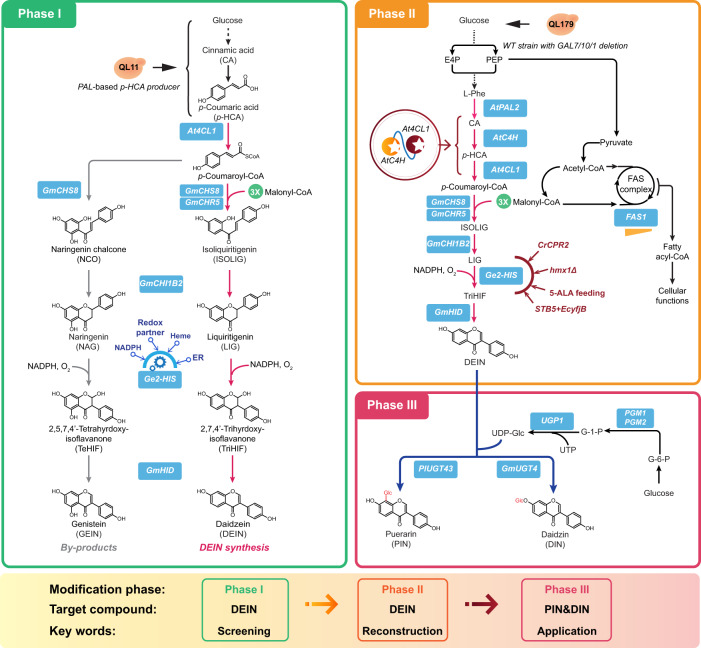
Engineering the de novo biosynthesis of isoflavonoid chemicals in yeast. A multi-phased metabolic engineering strategy was implemented to enable de novo production of isoflavonoids, following the pipeline of screening-reconstruction-application. In screening phase I (green box), a synthetic DEIN pathway was established and metabolic factors improving the performance of rate-limiting reactions were identified using a moderate *p*-HCA producing platform strain QL11. In reconstruction phase II (orange box), the DEIN production was further optimized in a wild-type strain QL179 harboring the deletion of galactose utilizing genes (*GAL7*/*10*/*1)*, through amplifying gene expression, enhancing substrate transfer, combining effective genetic targets identified in phase I and fine-tuning the expression of a key gene involved in competing metabolic pathway (orange triangle). For application phase III (magenta box), the generated DEIN platform strain was used as the starting point for the production of bioactive glucosides PIN and DIN through introducing plant glycosyltransferases and enhancing the supply of glycosyl group donor UDP-glucose. The selected plant biosynthetic genes and overexpressed yeast native genes for isoflavonoid production were highlighted in blue boxes. Magenta arrows, designed DEIN biosynthetic pathway. Blue arrows, reactions for generating glucosides; gray arrows, byproduct pathway. *At4CL1*, 4-coumarate-coenzyme A ligase 1 from *Arabidopsis thaliana*; *GmCHS8*, chalcone synthase from *Glycine max*; *GmCHR5*, chalcone reductase from *G. max*; *GmCHIB2*, chalcone isomerase from *G. max*; *Ge2-HIS*, 2-hydroxyisoflavanone synthase from *Glycyrrhiza echinata*; *GmHID*, 2-hydroxyisoflavanone dehydratase from *G. max*; *AtPAL2*, phenylalanine ammonia lyase from *A. thaliana*; *AtC4H*, cinnamic acid-4-hydroxylase from *A. thaliana*; *FAS1*, beta subunit of yeast fatty acid synthetase; *CrCPR2*, cytochrome P450 reductase from *Catharanthus roseus*; *STB5*, yeast native transcriptional factor; *EcyjfB*, NAD^+^ kinase from *Escherichia coli*; *GmUGT4*, isoflavone 7-*O*-glucosyltransferase from *G. max*; *PlUGT43*, isoflavone 8-*C*-glucosyltransferase from *Pueraria lobate*; *UGP1*, UDP-glucose pyrophosphorylase; *PGM1/2*, phosphoglucomutase 1/2. In addition, yeast heme degradation was disrupted by deleting heme oxygenase-coding gene *HMX1*. ER endoplasmic reticulum, E4P erythrose-4-phosphate, PEP phosphoenolpyruvate, L-Phe L-phenylalanine, 5-ALA 5-aminolevulinic acid, UDP-Glc uridine diphosphate-glucose, UTP uridine triphosphate, G-6-P glucose-6-phosphate, G-1-P glucose-1-phosphate.

Here we present the establishment of a de novo DEIN-producing yeast platform and its application for the biosynthesis of glycosylated isoflavonoids using a multi-phased metabolic engineering strategy (Fig. 1). In screening phase I, we first evaluated diverse plant enzymes to rebuild a functional DEIN pathway and extensively diagnosed exogenous and endogenous metabolic factors affecting the activity of key biosynthetic enzymes. Through pathway reconstruction in phase II, we improved the metabolic flux towards the DEIN pathway by implementing: (1) gene amplification to promote the expression of selected pathway genes; (2) protein fusion strategy to facilitate substrate trafficking; (3) additional genetic manipulations to increase the supply of metabolic cofactors (identified during phase I to be potential bottlenecks in pathway flux); (4) process development; and (5) fine-tuning of gene expression in the competing metabolic pathways. The systematic engineering enabled the production of 85.4 mg L^−1^ DEIN from glucose in shake flask cultivations. Finally, during application phase III, we demonstrated the efficient conversion of DEIN to bioactive glycosylated isoflavonoids by introducing plant glycosyltransferases. Supplementary Fig. 2 provides an overview of all strains constructed in the different phases of the development process.

## Results

### Phase I—Establishing the biosynthesis of scaffold isoflavone DEIN

In plants, the general phenylpropanoid pathway uses the aromatic amino acid (AAA) L-phenylalanine as a precursor for the biosynthesis of isoflavonoids as well as other flavonoids^24^. The initial steps engage phenylalanine ammonia lyase (PAL), cinnamic acid 4-hydroxylase (C4H), and 4-coumarate-coenzyme A ligase (4CL), resulting in the conversion of L-phenylalanine to *p*-coumaroyl thioester. Subsequently, the chalcone precursors, naringenin chalcone (NCO) and deoxychalcone isoliquiritigenin (ISOLIG), are synthesized from the condensation of *p*-coumaroyl CoA and three molecules of malonyl-CoA by chalcone synthase (CHS) alone or with the co-action of NADPH-dependent chalcone reductase (CHR), respectively^25^. Chalcone isomerase (CHI) is responsible for the further isomerization of chalcone to flavanone^26^. While naringenin (NAG) acts as the shared structural core in isoflavone GEIN and flavonoids pathways, the flavanone liquiritigenin (LIG) is used for the biosynthesis of isoflavone DEIN. The efficient generation of LIG represents therefore the first step towards developing a yeast platform for producing DEIN.

**Fig. 2:**
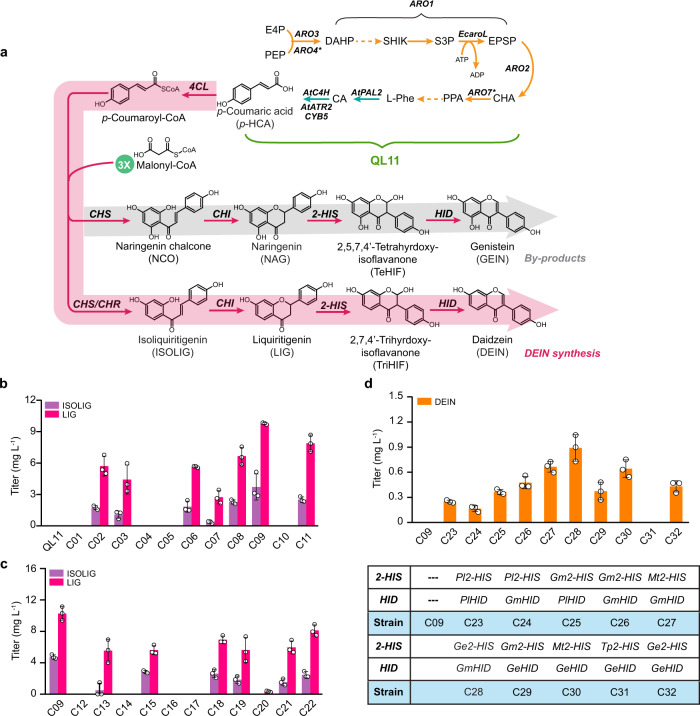
Building and validating the biosynthetic pathway for DEIN. **a** Schematic illustration of the biosynthetic pathways leading to the production of DEIN and related byproducts. To ensure efficient screening of biosynthetic enzymes for DEIN production, a *p*-HCA producing strain QL11, harboring overexpression of enzymes responsible for both endogenous (yellow arrows) and exogenous (blue arrows) reactions, was selected as the starting strain. *ARO3*, DAHP synthase; ARO4*, L-tyrosine-feedback-insensitive DAHP synthase (*ARO4*^*K229L*^); *ARO1*, pentafunctional aromatic protein; *EcaroL*, shikimate kinase from *E. coli*; *ARO2*, chorismate synthase; ARO7*, L-tyrosine-feedback-insensitive chorismate mutase (*ARO7*^*G141S*^); *AtATR2*, cytochrome P450 reductase from *A. thaliana*; CYB5, yeast native cytochrome b5. DAHP, 3-deoxy-D-arabino-2-heptulosonic acid 7-phosphate; SHIK, shikimate; S3P, shikimate-3-phosphate; EPSP, 5-enolpyruvyl-shikimate-3-phosphate; CHA, chorismic acid; PPA, prephenate. See Fig. 1 and its legend regarding abbreviations of metabolites and other gene details. **b**, **c** Production profiles of intermediates LIG and ISOLIG produced by yeast strains harboring different combinations of biosynthetic enzymes 4CL, CHS, CHR, and CHI. **d** DEIN production of C09 strain overexpressing different biosynthetic genes encoding 2-HIS and HID and relevant genetic characteristics of the resultant strains. For the source of selected plant genes: *Mt*, *Medicago truncatula*; *Tp*, *Trifolium pretense*. See Fig. 1 legend regarding abbreviations of other plant species. Cells were grown in a defined minimal medium with 30 g L^−1^ glucose as the sole carbon source, and cultures were sampled after 72 h of growth for metabolite detection. All data represent the mean of *n* = 3 biologically independent samples and error bars show standard deviation. The source data underlying figures (**b**−**d**) are provided in a Source Data file.

To facilitate the screening of biosynthetic enzymes for LIG production, we used a yeast platform strain (QL11) that has previously been reported to produce a moderate level of *p*-coumaric acid (*p*-HCA) (exceeding 300 mg L^−1^) from glucose without notable growth deficit^27^. The plant candidate genes have been selected according to their source and enzymatic specificity/activity. We first evaluated the combinations of candidate CHS, CHR, and CHI homologs, alongside the well-characterized At4CL1 from *Arabidopsis thaliana*, for the biosynthesis of LIG (Fig. 2a). Specifically, three CHS-coding genes, including leguminous *GmCHS8* (*Glycine max*) and *PlCHS* (*Pueraria lobate*) as well as non-leguminous *RsCHS* (*Rhododendron simsii*), were selected (Supplementary Fig. 3a). CHR activity has been mostly demonstrated in leguminous species^28^; thus *GmCHR5*, *PlCHR*, and *MsCHR* (*Medicago sativa*) were screened (Supplementary Fig. 3a). Plant CHIs can be categorized into distinct isoform groups according to their evolutionary path and enzymatic profiles. Whereas type I CHIs, common to all vascular plants, convert only NCO to NAG, legume-specific type II CHIs are capable of yielding both NAG and LIG^26^. Correspondingly, type II CHI-coding genes *PlCHI1* and *GmCHI1B2* were evaluated, together with a type I CHI-coding gene *PsCHI1* (*Paeonia suffruticosa*) being used as a control for enzymatic activity. All biosynthetic genes were chromosomally integrated and transcriptionally controlled by strong constitutive promoters. Co-overexpression of *At4CL1*, *GmCHR5*, *GmCHS8*, and *GmCHI1B2* resulted in the best LIG production at a level of 9.8 mg L^−1^ (strain C09) among all resultant strains (C01−C11, Fig. 2b). In addition, strains C01, C04−05 and C10 generated no detectable amounts of isoflavonoid intermediates, which could be attributed to the narrow substrate specificity of type I PsCHI1 (strain C01) and low enzymatic activity of PlCHR in yeast (strains C04-05 and C10), respectively (Fig. 2b and Supplementary Fig. 3b). Subsequently, individual gene replacement in strain C09 allowed the efficient screening of additional 4CL, CHS, and CHI variants. Five plant 4CL-coding genes, including *Pl4CL1*, *Gm4CL3*, *At4CL2*, *Ph4CL1* (*Petunia hybrida*), *Pc4CL2* (*Petroselinum crispum*) and a mutant *At4CL1m* (I250L, N404K, I461V)^29^, were overexpressed to generate LIG (strains C12−C17) (Supplementary Fig. 3a); however, none of these genes outperformed the wild-type *At4CL1* (C09) (Fig. 2c). Also, no improvement on LIG production was observed for strains expressing alternative variants of CHS (strains C18−C20) or CHI (strains C21−C22) (Fig. 2c and Supplementary Fig. 3c). Strain C09 was therefore chosen as the starting strain for further engineering to produce DEIN.

The entry point enzyme in the isoflavonoid biosynthetic pathway is 2-hydroxyisoflavanone synthase (2-HIS), which belongs to the cytochrome P450 family and catalyzes the intramolecular aryl migration of the B-ring yielding the intermediate 2-hydroxyisoflavanones^25^. Subsequently, dehydration of the resultant intermediate products, catalyzed by 2-hydroxyisoflavanone dehydratase (HID), gives rise to corresponding isoflavones^30^ (Fig. 2a). The 2-HIS and HID-coding genes were mainly identified in legumes that have been confirmed to produce isoflavonoids^25^. To identify efficient biosynthetic enzymes for DEIN formation, a group of leguminous 2-HIS and HID homologs were screened. Specifically, five 2-HIS-coding genes, including *Pl2-HIS*, *Gm2-HIS1*, *Mt2-HIS1* (*Medicago truncatula*), *Tp2-HIS* (*Trifolium pretense*), and *Ge2-HIS* (*Glycyrrhiza echinata*), and three HID-coding genes, including *PlHID*, *GmHID*, and *GeHID*, were combined and overexpressed in strain C09 (Fig. 2d). While most engineered strains generated detectable amounts of DEIN, strain C28, harboring the gene combination of *Ge2-HIS* and *GmHID*, accumulated the highest level of DEIN to 0.9 mg L^−1^ (Fig. 2d). These results show the feasibility to build de novo biosynthesis of DEIN in yeast by harnessing the diversity of plant pathway enzymes.

### Phase I—Engineering the redox partner of the key enzyme P450 2-HIS

Though strain C28 produced DEIN, only a low titer was obtained with a concomitant buildup of the biosynthetic intermediate LIG (Supplementary Fig. 4), suggesting inefficiencies in product formation in the later stage of the DEIN pathway. We, therefore, moved on to engineering the activity of Ge2-HIS, considering that the P450-mediated reactions are irreversible and often rate-limiting^31^ whereas HID is believed to facilitate the spontaneous dehydration of 2-hydroxyisoflavanones^16,30^.

**Fig. 3:**
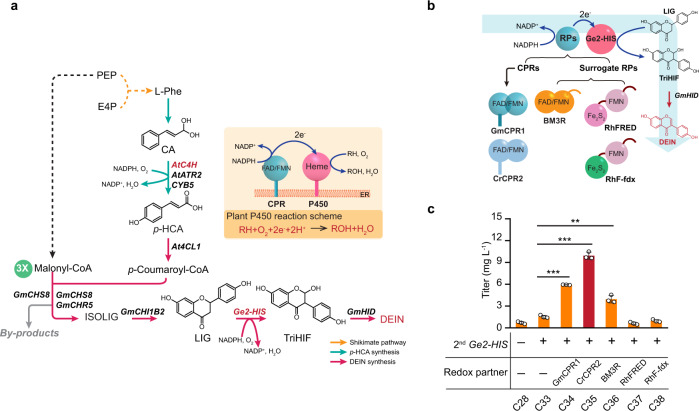
Tailoring the redox partner of Ge2-HIS for efficient DEIN production. **a** Schematic illustration of the biosynthetic pathways leading to the production of DEIN and related byproducts. P450 enzymes are indicated in magenta. In addition, a general catalytic mechanism of the membrane-bound plant P450 is shown in the inset. See Fig. 1 and its legend regarding abbreviations of metabolites and gene details. **b** Different redox partners (RPs) including CPR and surrogate redox partners from self-sufficient P450s were tested to enhance the catalytic activity of P450 Ge2-HIS. *GmCPR1*, cytochrome P450 reductase from *G. max*; BM3R, the eukaryotic-like reductase domain of P450BM3 from *Bacillus megaterium*; RhFRED, the FMN/Fe2S2-containing reductase domain of P450RhF from *Rhodococcus* sp. strain NCIMB 9784; RhF-fdx, a hybrid reductase by substituting Fe2S2 domain of RhFRED with ferredoxin (Fdx) from spinach. See Fig. 1 and its legend regarding abbreviations of metabolites and other gene details. **c** Effect of different RPs on the production of DEIN. Cells were grown in a defined minimal medium with 30 g L^−1^ glucose as the sole carbon source, and cultures were sampled after 72 h of growth for metabolite detection. Statistical analysis was performed by using Student’s *t* test (two-tailed; two-sample unequal variance; *p < 0.05, **p < 0.01, ***p < 0.001). All data represent the mean of *n* = 3 biologically independent samples and error bars show standard deviation. The source data underlying panel (**c**) are provided in a Source Data file.

The redox partner (RP) is an integral part of canonical P450 systems that shuttles the electrons derived from NAD(P)H to the heme iron-center to enable oxygen cleavage and substrate monooxygenation^32^. The endoplasmic reticulum (ER)-anchored plant P450s recruit a single RP protein, the membrane-attached flavin adenine dinucleotide (FAD)/flavin mononucleotide (FMN)-containing cytochrome P450 reductase (CPR), to transfer electrons^33^ (Fig. 3a). Co-expression of the cognate CPRs is a common practice for reconstituting P450-involved plant biosynthetic pathways in yeast, such as the production of terpenoid^12^ and alkaloid^11^, which has been believed to permit more efficient P450-CPR coupling compared to the native yeast CPR^33^. In addition, plant CPRs exhibit a certain degree of versatility, due to the high level of conservation represented by the amino acid residues that mediate P450 interactions^33^. Accordingly, in our DEIN-producing strains, Ge2-HIS might receive electrons from AtATR2, a CPR homolog of *A. thaliana* introduced for optimizing the activity of P450 AtC4H to produce *p*-HCA (Fig. 3a). However, the limiting Ge2-HIS activity evidenced by a low DEIN titer of strain C28 motivated us to exploit alternative plant CPRs, including *GmCPR1* and *CrCPR2* (*Catharanthus roseus*) (Fig. 3b). The selected CPR-coding genes were chromosomally integrated in combination with a second copy of *Ge2-HIS* to strain C28. Using this approach, DEIN production of both *GmCRP1*-expressing strain C34 and *CrCRP2*-expressing strain C35 was significantly enhanced to a titer of 5.9 and 9.9 mg L^−1^, respectively, accounting for a 284 and 544% increase compared with that of strain C33 only harboring a second copy of *Ge2-HIS* (Fig. 3c). This strongly indicates that there may be an improved coupling of the alternative CPRs to Ge2-HIS.

Among the diverse range of P450-dependent electron transport systems, an intramolecular electron transfer mechanism has been employed by the so-called self-sufficient P450s, in which the RP domain is naturally fused with the catalytic domain^32^. This unique group of P450s exhibit many advantages, including higher electron transfer efficiency, improved substrate turnover, as well as no need for a separate RP, and therefore are of great interest for biotechnological applications^34^. To mimic this intramolecular electron transfer, surrogate RPs derived from prominent self-sufficient bacterial P450s, including (1) BM3R, the eukaryotic-like reductase domain of P450BM3 from *Bacillus megaterium*^35^; (2) RhFRED, the FMN/Fe_2_S_2_-containing reductase domain of P450RhF from *Rhodococcus* sp. strain NCIMB 9784^36^; and (3) RhF-fdx, a hybrid reductase by substituting Fe_2_S_2_ domain of RhFRED with ferredoxin (Fdx) from spinach^37^, were C-terminally fused to Ge2-HIS and investigated for DEIN production (Fig. 3b). These chimeras were co-expressed with an additional copy of *Ge2-HIS*. While strain C36 harboring Ge2-HIS/BM3R fusion had a 156% increase in DEIN production, compared with the control strain C33 (Fig. 3c), the introduction of RhFRED and RhF-fdx decreased the biosynthesis of DEIN of corresponding engineered strains C37 and C38 (Fig. 3c).

### Phase I—Identifying potential metabolic factors improving 2-HIS activity

**Fig. 4:**
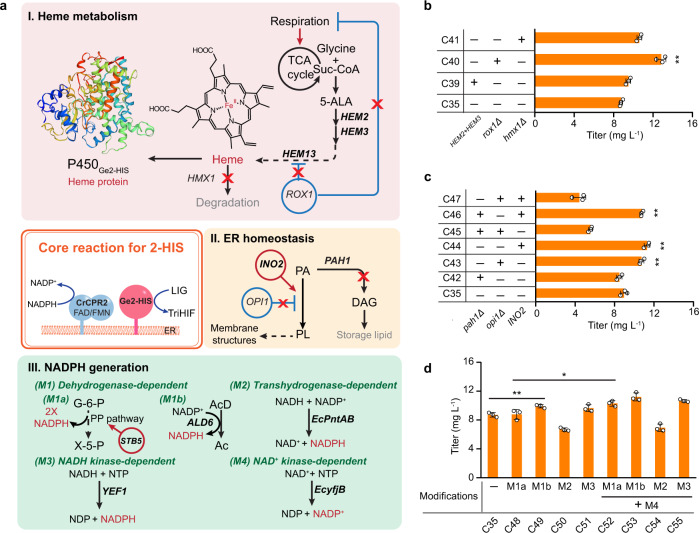
Investigation of metabolic factors affecting DEIN biosynthesis. **a** Schematic illustration of the genetic modifications performed to relieve potential metabolic limitations, regarding heme metabolism (I), ER homeostasis (II), and cofactor NADPH generation (III), to improve the performance of P450 Ge2-HIS. Overexpressed genes are shown in bold and gene deletion is marked with a red cross. Red circle and arrows, transcriptional factors (TFs) activating a metabolic gene/pathway; Blue circle and flat-head arrow, TFs repressing a metabolic gene/pathway. *HEM2*, 5-aminolevulinic acid dehydratase; *HEM3*, 4-porphobilinogen deaminase; *HEM13*, coproporphyrinogen III oxidase; *ROX1*, heme-dependent repressor of hypoxic genes; *PAH1*, phosphatidate phosphatase; *INO2*/*OPI1*, transcription activator/repressor of phospholipid biosynthetic genes; *ALD6*, cytoplasmic NADP^+^-dependent aldehyde dehydrogenase; *EcpntAB*, membrane-bound transhydrogenase from *E. coli*; *YEF1*, ATP-NADH kinase. Suc-CoA, succinyl-CoA; PA, phosphatidic acid; PL, phospholipid; DAG, diacylglycerol; X-5-P, xylulose-5-phosphate; AcD, acetaldehyde; Ac, acetate; NTP, nucleoside triphosphates; NDP, nucleoside diphosphates. See Fig. 1 and its legend regarding abbreviations of metabolites and other gene details. Production of DEIN by engineered yeast strains derived from strategy groups I (**b**), II (**c**), and III (**d**), respectively. Cells were grown in a defined minimal medium with 30 g L^−1^ glucose as the sole carbon source, and cultures were sampled after 72 h of growth for metabolite analysis. Statistical analysis was performed by using Student’s *t* test (two-tailed; two-sample unequal variance; *p < 0.05, **p < 0.01, ***p < 0.001). All data represent the mean of *n* = 3 biologically independent samples and error bars show standard deviation. The source data underlying panels (**b**−**d**) are provided in a Source Data file.

Besides pairing with an appropriate RP, the attainment of high P450 catalytic efficiency is challenged by (I) the intracellular level of heme for the assembly of holoenzymes, (II) the ER microenvironment to accommodate functional membrane proteins and (III) the availability of redox cofactors. Next, we moved to uncover potential bottlenecks regarding these factors in limiting the biosynthesis of DEIN (Fig. 4a).

The production of active P450s requires sufficient incorporation of cofactor heme, which may deplete the intracellular pool of heme and thereby incur a cellular stress response that in turn damages the net enzymatic activity^38^. To mitigate this potential adverse effect on the activity of Ge2-HIS, we tested different approaches to regulate heme metabolism of yeast (Fig. 4a-I). Co-overexpression of two rate-limiting enzymes in yeast heme biosynthesis, encoded by genes *HEM2* and *HEM3*^39^, slightly enhanced the production of DEIN to 9.5 mg L^−1^ (strain C39, Fig. 4b). In addition, a previous study illustrated that inactivation of the transcriptional repressor Rox1 could render an elevated cellular heme level^40^, resulting from the derepression of the heme biosynthetic gene *HEM13*. We, therefore, deleted *ROX1* in strain C35, yielding a DEIN titer of 12.8 mg L^−1^ by the resultant strain C40 (Fig. 4b), a 46% increase compared with that of the parental strain. Besides reinforcing the biosynthetic pathway, reducing degradation of heme also contributes to its intracellular accumulation and improves the P450s activity^41^. Accordingly, upon the deletion of *HMX1*, which encodes heme oxygenase responsible for heme degradation, the production of DEIN of the resultant strain C41 (10.6 mg L^−1^) was increased by 21% relative to strain C35 (Fig. 4b).

Most plant-derived P450s and CPRs are independently tethered onto the ER via hydrophobic transmembrane anchors^42^. Modulating the biogenesis and size of the ER has previously been shown to enhance P450-involved biosynthesis of terpenoids in *S. cerevisiae*^43,44^, a result which is likely due to a higher protein folding capacity enabled by ER expansion. To evaluate the possible beneficial effect of ER expansion for DEIN biosynthesis, we therefore elevated the intracellular level of phospholipids for ER assembly by implementing (1) the deletion of *PAH1*-encoded phosphatidate phosphatase that competes for the phospholipid precursor^45,46^; (2) the deletion of the transcription factor Opi1 and (3) overexpression of the transcription factor Ino2 that negatively and positively control the expression of UAS_INO_-containing phospholipid biosynthetic genes, respectively^47^ (Fig. 4a-II). A significantly enhanced DEIN generation was observed for the *OPI1* deletion strain C43 (10.8 mg L^−1^) and the *INO2*-overexpressing strain C44 (11.3 mg L^−1^), representing a 20 and 26% increase relative to strain C35 (Fig. 4c). Moreover, strain C46 harboring *PAH1* deletion and *INO2* overexpression also had a 20% increase in DEIN formation (10.8 mg L^−1^) compared with that of strain C35, whereas other combinatorial modifications led to a reduction in DEIN titers of strains C45 and C47 (Fig. 4c). This difference could be attributed to the remarkably impaired cell growth of the latter two strains (Supplementary Fig. 5).

Redox cofactors NAD(P)H, the ultimate electron source in cellular metabolism, are indispensable for the catalytic cycle of plant P450s^33^. Lack of NAD(P)H could reduce the P450 activity due to inefficient electron transfer. A recent report indicated that improved cellular NADPH level could enhance the P450-mediated protopanaxadiol production^48^. Thus, we decided to reroute the redox metabolism to fuel the activity of Ge2-HIS. In the first strategy, genetic modifications engaged to boost the direct generation of NADPH were devised and individually implemented, including (M1a) overexpression of the transcriptional factor Stb5 that activates the expression of genes involved in the pentose phosphate pathway (PPP)^49^, the major source of NADPH for anabolic processes in yeast; (M1b) overexpression of the *ALD6*-encoded cytoplasmic NADP^+^-dependent aldehyde dehydrogenase that converts acetaldehyde to acetate; (M2) introduction of *E. coli pntAB* genes encoding a membrane-bound transhydrogenase capable of reducing NADP^+^ at the expense of NADH^50^; and (M3) overexpression of yeast *YEF1*-encoded ATP-NADH kinase that directly phosphorylates NADH to NADPH^51^, resulting in an elevated concentration of the phosphorylated form of this cofactor without impact on the NADPH/NAPD^+^ ratio (Fig. 4a-III). The resultant strains C49 (M1b) and C51 (M3) produced 9.9 and 9.7 mg L^−1^ of DEIN, representing a 14% and 11% increase, respectively, compared with the parental strain C35 (Fig. 4d). Moreover, combined overexpression of NAD^+^ kinase (NADK), the sole enzyme leading to de novo NADP^+^ biosynthesis, with an NADP^+^ reducing enzyme was shown to improve NADPH-consumed bacterial isobutanol production^52^. We, therefore, evaluated this strategy via further co-expressing a prokaryotic NADK-coding gene *EcyfjB* (M4) (Fig. 4a-III). The reported synergistic effect was most evident for strain C52, containing (M1a) and (M4), which had a 17% increase in DEIN titer relative to strain C48 (Fig. 4d).

### Phase II—Gene amplification and engineering of substrate trafficking improve DEIN biosynthesis

In screening phase I, through performing combinatorial gene screening in parallel with multiple genetic modifications, we achieved substantial de novo DEIN biosynthesis and identified important metabolic factors affecting its overproduction in yeast. However, the resultant strains exhibited two major unfavorable phenotypes, including a large amount of non-consumed precursor *p*-HCA (Supplementary Fig. 6) and the formation of several metabolic intermediates and byproducts (Supplementary Fig. 7). This may result from (1) metabolic imbalance between upstream *p*-HCA producing and the downstream pathways, (2) insufficient activity and (3) substrate promiscuity of some of the plant enzymes, and (4) inefficient cytosolic substrate transfer. We therefore next aimed to improve the production of DEIN via relieving these potential metabolic barriers.

**Fig. 5:**
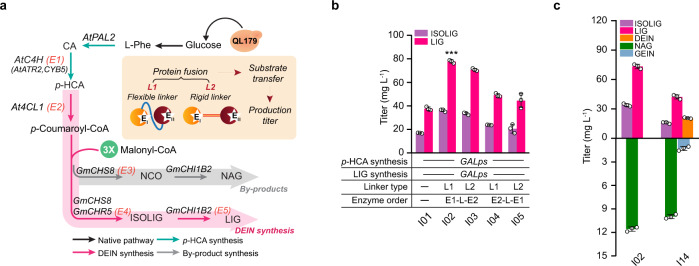
Gene amplification and engineering of substrate trafficking improve DEIN production. **a** Schematic view of the targets and strategies to improve the substrate transfer along the DEIN biosynthetic pathway. Two different oligopeptide linkers (flexible linker L1, GGGS; rigid linker L2, VDEAAAKSGR) were employed to fuse the adjacent metabolic enzymes. Strain QL179 was selected to implement *GAL* promoters (*GALps*)-mediated gene amplification. See Fig. 1 and its legend regarding abbreviations of metabolites and other gene details. **b** Quantification of metabolic intermediates produced by strains carrying a fused enzyme of AtC4H (E1) and At4CL1 (E2). **c** Comparison of the production profiles between parental strain I02 and I14 harboring additional overexpression of selected metabolic enzymes Ge2-HIS and GmHID and auxiliary CrCPR2. Cells were grown in a defined minimal medium with 30 g L^−1^ glucose as the sole carbon source and 10 g L^−1^ galactose as the inducer. Cultures were sampled after 72 h of growth for metabolite detection. Statistical analysis was performed by using Student’s *t* test (two-tailed; two-sample unequal variance; *p < 0.05, **p < 0.01, ***p < 0.001). All data represent the mean of *n* = 3 biologically independent samples and error bars show standard deviation. The source data underlying panels (**b**, **c**) are provided in a Source Data file.

To reduce the metabolic loss due to an excessive supply of *p*-HCA in background strain QL11, we instead turned to reconstructing the DEIN biosynthesis in a “clean” background without an engineered AAA pathway. With this strain, we used the galactose-induced dynamic transcription mechanism^53^, which is mediated by *GAL* promoters (*GALps*) and has been successfully used for high-level production of value-added chemicals^12,27^, to enhance the expression of DEIN pathway genes. A previously described strain QL179^27^, derived from IMX581 by disrupting galactose utilization genes *GAL1*/*10*/*7*, was thus selected to confer galactose as the gratuitous inducer to activate *GALps* (Fig. 5a). As expected, simultaneous introduction of the *GALps*-controlled *p*-HCA and LIG pathways greatly boosted LIG titer to 37.6 mg L^−1^ in strain I01 (Fig. 5b), a 284% increase relative to strain C09, in which a constitutive gene expression pattern was used. Interestingly, a markedly low level of *p*-HCA was detected in strain I01, indicating that to a certain extent the metabolic flux between the redesigned *p*-HCA producing and consuming pathways was balanced (Supplementary Fig. 8).

The spatial organization of catalytic enzymes is increasingly recognized to be critical for optimizing the metabolic flow through multi-step biosynthetic pathways^54^. Coordinating the physical distance of adjacent enzymes, mediated by peptide linkers or synthetic scaffolds, could elevate local concentrations of enzymes and metabolites thereby speeding up the turnover of intermediates, minimizing metabolic crosstalk, and improving reaction flux^55^. To test this, we implemented a synthetic fusion enzyme strategy to optimize the substrate trafficking through the LIG pathway. Specifically, two distinct oligopeptide linkers possessing flexible (GGGS, L1) or rigid (VDEAAAKSGR, L2) conformations^56^ were applied to fuse the coding sequences of neighboring enzymes (Fig. 5a). Fused AtC4H (E1) with At4CL1 (E2) in both tandem orientations together with other biosynthetic genes were chromosomally integrated into strain QL179, creating strains I02−I05 (Fig. 5b). A significantly enhanced LIG production was observed for these strains; and fusion enzyme in the E1-L1-E2 orientation provided the highest LIG titer of 77.7 mg L^−1^ (strain I02), representing a 107% improvement compared with strain I01 (Fig. 5b). This result indicates that the physical fusion of two enzymes responsible for the formation and consumption of *p*-HCA, respectively, could greatly drive the metabolic flux towards LIG biosynthesis. Encouraged by this, we further introduced fusion enzymes consisting of GmCHS8 (E3)-Linker-GmCHR5 (E4) or GmCHR5 (E4)-Linker-GmCHI1B2 (E5) to strain I02 (Fig. 5a). In contrast, here we found the titer of LIG to be decreased in the resultant strains I06-I13 (Supplementary Fig. 9), which may be attributed to a reduced CHR activity of fusion enzymes, considering the lower variation we observed for byproduct formation. We, therefore, selected strain I02 as the platform for incorporating the best downstream DEIN-forming biosynthetic and auxiliary enzymes (Ge2-HIS, GmHID, and CrCPR2). The DEIN titer of resultant strain I14 (20.7 mg L^−1^, Fig. 5c) doubled relative to strain C35 harboring the constitutive promoter-driven DEIN pathway (9.9 mg L^−1^, Fig. 3c).

### Phase II—Combinatorial strategies to increase DEIN production

**Fig. 6:**
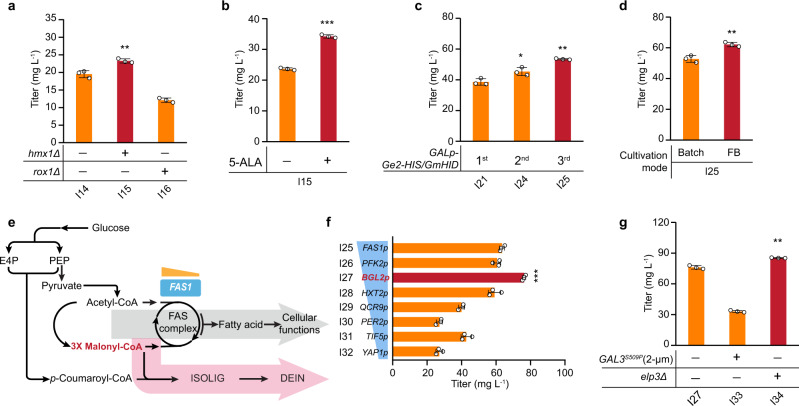
Combinatorial optimization to increase the production of DEIN. **a** Effect of deleting genes involved in the regulation of heme metabolism on DEIN biosynthesis. Production of DEIN by strains fed with the heme biosynthetic precursor 5-ALA (**b**) or expressing different copies of *Ge2-HIS* and *GmHID* genes (**c**). **d** Process optimization for DEIN production. Cells were grown in a defined minimal medium with 30 g L^−1^ glucose (batch) or with six tablets of FeedBeads (FB) as the sole carbon source and 10 g L^−1^ galactose as the inducer. Cultures were sampled after 72 h (batch) or 90 h (FB) of growth for metabolite analysis. **e** Schematic view of the interplay between isoflavonoid biosynthesis and yeast cellular metabolism connected by the branchpoint malonyl-CoA. See Fig. 1 and its legend regarding abbreviations of metabolites and gene details. **f** Fine-tuning the expression of gene *FAS1* via promoter engineering improves DEIN formation under optimized cultivation conditions. **g** Effect of genetic modifications altering the regulation of *GAL* induction on DEIN production under optimized cultivation conditions. The constitutive mutant of galactose sensor Gal3 (*GAL3*^*S509P*^) was overexpressed from a multi-copy plasmid (2 µm) under the control of *GAL10p* and gene *ELP3*, encoding a histone acetyltransferase, was deleted. Cells were grown in a defined minimal medium with six tablets of FB as the sole carbon source and 10 g L^−1^ galactose as the inducer. Cultures were sampled after 90 h of growth for metabolite detection. Statistical analysis was performed by using Student’s *t* test (two-tailed; two-sample unequal variance; *p < 0.05, **p < 0.01, ***p < 0.001). All data represent the mean of *n* = 3 biologically independent samples and error bars show standard deviation. The source data underlying panels (**a**−**d**) and (**f**, **g**) are provided in a Source Data file.

Improving the expression of biosynthetic genes and the cellular substrate transfer greatly enhanced the DEIN titer of strain I14. However, we also observed considerable accumulation of both intermediates (15.8 mg L^−1^ of ISOLIG and 42.3 mg L^−1^ of LIG, Fig. 5c) as well as byproducts (10.0 mg L^−1^ of NAG and 1.3 mg L^−1^ of GEIN, Fig. 5c), showing a need for strengthening the later stage of DEIN biosynthesis. To solve this, we first aimed to improve the activity of Ge2-HIS by combining effective P450-centered genetic targets identified in phase I engineering (Fig. 4a). Expectedly, the removal of heme degradation by disrupting *HMX1* gene resulted in a 19% increase in DEIN titer of strain I15 (23.3 mg L^−1^) compared with that of strain I14 (Fig. 6a), whereas *ROX1* deletion negatively affected DEIN production (strain I16, Fig. 6a), this potentially being caused by the resulting loss of its regulatory role in stress resistance of *S. cerevisiae*^40^. Subsequently, the deletion of *OPI1* or overexpression of *INO2* genes was individually carried out to stimulate ER expansion in strain I15; however, both resultant strains gave a lower DEIN titer (Supplementary Fig. 10a). While compromised cell growth associated with these strains (Supplementary Fig. 10b) could have weakened their DEIN generation, a shortage of intracellular heme may also be limiting the functional P450 folding and thereby blunting the effect of ER adjustment. Previous studies showed that feeding 5-aminolevulinic acid (5-ALA), the direct precursor of heme biosynthesis, could significantly increase the cellular heme level of yeast^38^. Indeed, we found exogenous supplementation of 1 mM 5-ALA resulted in 45% (34.3 mg L^−1^, strain I15 + A), 65% (17.3 mg L^−1^, strain I17 + A), and 42% (27.1 mg L^−1^, strain I18 + A), respectively, further increases in DEIN production for the strains tested (Fig. 6b and Supplementary Fig. 10a). ER-targeting modifications however exhibited no beneficial effects on DEIN production, which could be ascribed to the distinct engineering context of strains C35 and I15, implying a need for fine-tuning the interplay between ER biogenesis and P450 anchoring. Thus, strain I15 was subject to the integration of NADPH generation systems. Among selected targets, co-overexpression of native *STB5* and bacterial *EcyfjB* genes (M1a + M4) led to the highest DEIN titer of 40.2 mg L^−1^, a 12% improvement relative to strain I15 (strain I21, Supplementary Fig. 11).

Based on established results of cofactor refinement, we speculated that the availability of biosynthetic enzymes could emerge as a limiting factor for the conversion of LIG to DEIN. Especially, previous reports indicated that the 2-HIS enzyme in microsomal preparation from soybean cells is labile^57^ and the catalytic characteristics of 2-HIS have evolved by sacrificing protein stability^58^. We, therefore, introduced extra copies of the best DEIN-forming gene combination, *Ge2-HIS* with *GmHID*, to strain I21. Interestingly, while there was a 17% increase in DEIN production in strain I24 containing the second copy of selected genes, the introduction of the third copy of this gene combination further enhanced DEIN production to 53.5 mg L^−1^ (strain I25), representing a 38% increase compared with strain I21 (Fig. 6c).

Compared with batch (glucose excess) cultivations, yeast cells grown under glucose-limited cultivation are known to have a higher biomass yield and an enhanced PPP flux^59^, the latter being anticipated to favor AAA biosynthesis by increasing the availability of the precursor erythrose 4-phosphate. We, therefore, grew DEIN-producing strains under a mimicked glucose-limited fed-batch cultivation by using FeedBeads (FB) (Supplementary Fig. 12), a slow-release system for glucose^60^. Expectedly, under FB conditions, strain I25 produced 62.1 mg L^−1^ of DEIN, representing an 18% increase relative to the same strain under batch conditions (Fig. 6d). Moreover, the application of this FB strategy led to observable growth improvements and a striking increase in byproduct formation of strain I25 (Supplementary Fig. 13). These results agree also with our previous work wherein significant improvements on cellular biomass formation and *p*-HCA production could be achieved by growing yeast cells under glucose-limited conditions^27^.

For the biosynthesis of one molecule of DEIN, one molecule of *p*-coumaroyl-CoA and three molecules of malonyl-CoA are consumed (Fig. 6e). Following our optimization of metabolic flux using the *p*-HCA pathway and reinforcement of the DEIN biosynthetic pathway, we speculated that the supply of malonyl-CoA had become the next limiting factor in DEIN production. In *S. cerevisiae*, the majority of cytosolic malonyl-CoA pool is invested in the synthesis of fatty acids (FAs), which are essential for multiple cellular functions and cell growth^61^. The FAS complex, composed of Fas1 and Fas2, is responsible for FAs generation in yeast with the *FAS1* gene product known to impose positive autoregulation on *FAS2* expression to coordinate the activity of the FAS complex^62^. Hence, we set out to fine-tune the expression of the *FAS1* gene to divert malonyl-CoA towards DEIN biosynthesis (Fig. 6e). A group of yeast promoters, exhibiting differential transcriptional activities in response to glucose^63^ (Supplementary Table 1), were used to substitute the native *FAS1* promoter. Among seven evaluated promoters, replacement with *BGL2p* brought about the greatest DEIN titer of 76.3 mg L^−1^ (strain I27), a 20% increase compared with strain I25 (Fig. 6f). Additionally, the production of intermediates and byproducts was also notably elevated (Supplementary Fig. 14), further reflecting that promoter replacement of *FAS1* has boosted the overall metabolic flux towards isoflavonoids.

The galactose-induced transcriptional response (the *GAL* induction) of *S. cerevisiae* initiates with the association of the galactose sensor Gal3 with the regulatory inhibitor Gal80, leading to dissociation of the latter from the transcription activator Gal4, thereby allowing rapid expression of *GAL* genes^53^. Constitutive *GAL3* mutants (*GAL3*^*c*^) have been demonstrated to confer galactose-independent activation of Gal4 ^64^. This trait was recently engineered to build a positive feedback genetic circuit in which expressed Gal3^c^ provokes greater expression of Gal3^c^ and thereby enhances *GAL* induction^65^. We speculated that DEIN production may benefit from overexpression of such a Gal3^c^ mutant as a result of further induction of the *GALps*-controlled biosynthetic pathway. However, when expressed from a high-copy vector under the control of *GAL10p*, the introduction of constitutive Gal3^S509P^ mutant led to a significant decrease in both DEIN and GEIN titers (Fig. 6g and Supplementary Fig. 15). On the other hand, by deleting gene *ELP3*, encoding a histone acetyltransferase that is part of elongator and RNAPII holoenzyme^66^, a final DEIN titer of 85.4 mg L^−1^ was achieved in the resultant strain I34 (Fig. 6g), representing a 12% improvement relative to strain I27. The production of GEIN was also slightly increased to 33.7 mg L^−1^ (Fig. 6g and Supplementary Fig. 15). These results also show to be consistent with a published study wherein *ELP3* deletion was found to enhance the *GAL1p*-mediated beta-galactosidase activity in the presence of galactose^67^. The high-level accumulation of DEIN could exert cellular toxicity in *S. cerevisiae* and thereby impede the further improvement of its titer. We, therefore, evaluated the growth profiles of the background strain IMX581 under different concentrations of DEIN within its solubility limit. The results revealed that yeast could tolerate up to 150 mg L^−1^ of DEIN without significant loss of growth capacity (Supplementary Fig. 16). Hence, it is reasonable to assume that the production of DEIN is non-toxic to yeast at the levels produced here.

### Phase III—Production of DEIN-derived glucosides

Glycosylation represents a prevalent tailoring modification of plant flavonoids that modulates their biochemical properties, including solubility, stability, and toxicity^68^. In soybean, enzymatic 7-*O*-glucosylation of DEIN leads to the biosynthesis of DIN^69^, one of the key ingredients found in soybean-derived functional foods and nutraceuticals^70^. Moreover, puerarin (PIN), an 8-*C*-glucoside of DEIN, is ascribed as the major bioactive chemical of *P. lobate* roots extract, which has long been used in Chinese traditional medicine for the prevention of cardiovascular diseases^71^. Recent studies also show that PIN exhibits diverse pharmacological properties including antioxidant, anticancer, vasodilation, and neuroprotection-related activity^72^. With the establishment of efficient DEIN-producing yeast platform during reconstruction phase II (Fig. 6g), we explored its application potential in the production of PIN and DIN.

**Fig. 7:**
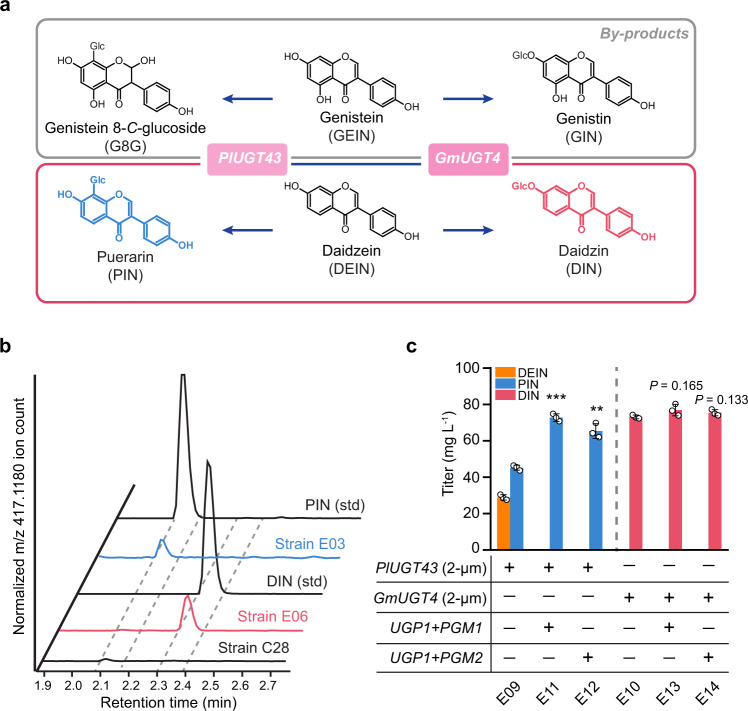
Production of DEIN-derived glucosides. **a** Schematic view of the engineered metabolic pathways for the biosynthesis of glucosides PIN and DIN (pink box), and relevant byproducts (gray box). See Fig. 1 legend for gene details. **b** Characterization of metabolic enzymes responsible for glucoside biosynthesis. Three copies of *PlUGT43* and *GmUGT4* under the control of constitutive promoters were integrated into the DEIN producer C28, resulting in strains E03 and E06, respectively. Cells were grown in a defined minimal medium with 30 g L^−1^ glucose as the sole carbon source, and cultures were sampled after 72 h of growth for LC-MS analysis. **c** Production profiles of PIN and DIN in DEIN hyper-producing strain I34 background with or without increased UDP-glucose supply. Combined overexpression of genes *PGM1*/*2* with *UPG1* was implemented to enhance the generation of glycosyl group donor UDP-glucose. See Fig. 1 legend for gene details. Cells were grown in a defined minimal medium with six tablets of FB as the sole carbon source and 10 g L^−1^ galactose as the inducer. Cultures were sampled after 90 h of growth for metabolite detection. Statistical analysis was performed by using Student’s *t* test (two-tailed; two-sample unequal variance; *p < 0.05, **p < 0.01, ***p < 0.001). All data represent the mean of *n* = 3 biologically independent samples and error bars show standard deviation. The source data underlying figure **c** are provided in a Source Data file.

The biosynthesis of flavonoid glycosides is mediated by UDP-sugar-glycosyltransferases (UGTs), which catalyze the formation of *O*−*C* or *C*−*C* bond linkages between the glycosyl group from uridine diphosphate (UDP)-activated donor sugars and the acceptor molecules^1,73^. While a soybean isoflavone 7-*O*-glucosyltransferase exhibiting broad substrate scope was first described over 10 years ago^69^, only recently Funaki et al.^74^ revealed that its homolog GmUGT4 enables highly specific 7-*O*-glucosylation of isoflavones. On the other hand, the complete PIN pathway was fully elucidated when Wang et al.^71^ successfully cloned and functionally characterized a *P. lobata* glucosyltransferase, encoded by *PlUGT43*, which displays strict in vitro 8-*C*-glucosylation activity towards isoflavones and enables PIN production in *PlUGT43*-expressed soybean hairy roots. We, therefore, tested the feasibility of using these UGTs in generating DEIN glucosides (Fig. 7a). Different copies of *PlUGT43* and *GmUGT4* under the control of constitutive promoters were integrated into the basic DEIN producer C28, but the resultant yeast strains (E01−E03 for PIN and E04−E06 for DIN, Supplementary Fig. 2) generated no detectable level of glycosides for HPLC analysis. However, through further analysis with high-resolution LC-MS, we validated that strains E03 and E06 could generate trace amount of PIN and DIN, respectively (Fig. 7b and Supplementary Fig. 17), demonstrating that both UGTs were functional in yeast.

Besides the selection of active UGTs, the supply of glycosyl group donor UDP-glucose also plays a pivotal role in regulating glucoside production. With the efficient DEIN producer I34 in hand, we moved to enhance its capacity for biosynthesizing UDP-glucose. In *S. cerevisiae*, metabolic enzymes phosphoglucomutase (encoded by *PGM1* and *PGM2*) and UDP-glucose pyrophosphorylase (encoded by *UGP1*) catalyze the formation of UDP-glucose branching from glucose-6-phosphate (Supplementary Fig. 18a). Through chromosomally integrated expression of *UGP1* with *PGM1* or *PGM2* in strain I34, strains E07 and E08 were created. Additionally, to ensure adequate UGTs activity, two multi-copy plasmids, harboring genes *PlUGT43* (pQC229) and *GmUGT4* (pQC230) under the control of *GAL1p*, were constructed and individually introduced into the high-level producers of DEIN (strains I34, E07, and E08). In doing so, we found the resultant strains E09 and E10, derived from the I34 background, to produce 45.2 mg L^−1^ of PIN and 73.2 mg L^−1^ of DIN, respectively (Fig. 7c). Interestingly, compared with strain E10, the *PlUGT43*-expressing strain E09 still accumulated a considerable amount of DEIN (28.9 mg L^−1^, Fig. 7c). This discrepancy may be attributed to the insufficient activity of PlUGT43, whose determined kinetic parameters for DEIN (*K*_cat_ = 0.35 s^−1^, *K*_m_ = 32.8 µM, and *K*_cat_/*K*_m_ = 1.1 × 10^4^ M^−1^ s^−1^)^71^ show to be considerably less optimal compared to GmUGT4 (*K*_cat_ = 5.89 s^−1^, *K*_m_ = 20.3 µM, and *K*_cat_/*K*_m_ = 2.91 × 10^5^ M^−1^ s^−1^)^74^. Furthermore, the conversion of GEIN to 25.9 mg L^−1^ of *C*-glycoside genistein 8-*C*-glucoside (G8G) and 26.5 mg L^−1^ of *O*-glycoside genistin (GIN) was observed for strains E09 and E10 (Supplementary Fig. 18b and c), respectively, since the selected UGTs exhibit comparable glycosyltransferase activity towards GEIN^71,74^. Moreover, the overexpression of UDP-glucose-forming genes resulted in full consumption of DEIN and enhanced PIN production to 72.8 mg L^−1^ in E07-derived strain E11 and 65.4 mg L^−1^ in E08-derived strain E12, representing a 61% and 45% increase respectively compared with strain E09 (Fig. 7c). On the other hand, such modifications resulted in no significant increase in the production of DIN (Fig. 7c) and byproduct glucosides (Supplementary Fig. 18b and c), reflecting a shortage of precursor isoflavones. Similarly, we analyzed the growth-inhibitory effects of the two glucosides on strain IMX581. Compared with their aglycon DEIN, an increased level of PIN (500 mg L^−1^) and DIN (250 mg L^−1^) can be tolerated by yeast to retain normal cell growth (Supplementary Fig. 19); both concentrations are much higher than the best titer achieved for the two glucosides in our study. Specifically, supplementation of DIN improved growth of yeast, which could result from the uptake of DIN and then release of glucose catalyzed by native glucosidases, such as the steryl-beta-glucosidase Egh1p^75^.

## Discussion

Isoflavonoids play important roles in the plant defense system and have many human health-related benefits. They, therefore, represent promising candidates in the development and engineering of agents for agricultural, nutraceutical, and pharmaceutical applications. Here we established a yeast-based de novo production platform for the efficient production of the isoflavonoid carbon skeleton DEIN as well as the high-value glucosides PIN and DIN. This was achieved by first identifying functional biosynthetic enzymes to generate DEIN (screening phase I), then by optimizing metabolic flux at enzyme and pathway levels to further increase DEIN titer (reconstruction phase II) and finally by introducing plant UGTs to convert DEIN to corresponding glucosides (application phase III).

Gene duplication and diversification occur in the evolution of plant secondary metabolism to tackle the changing environment, creating a rich variability and complexity of plant products as a result^76^. However, this functional divergence poses an obstacle to identifying ideal candidate enzymes for reconstructing heterologous biosynthetic pathways for plant metabolite production. Most of the structural genes involved in isoflavonoid pathways have been characterized^25^, and here we exploited their genetic diversity, by performing a combinatorial evaluation of biosynthetic genes from both leguminous and non-leguminous plants, to enable DEIN production (Fig. 2b–d). The P450s constitute the most versatile tailoring enzymes that catalyze irreversible and often rate-limiting reactions in the biosynthesis of plant-specialized products^31^. Though *S. cerevisiae* is generally identified as a superior host for the functional expression of membrane-bound plant P450s over its prokaryotic counterparts, extra efforts are required to maximize their catalytic efficiency. Two distinct P450s, the upstream C4H hydroxylating cinnamic acid and the downstream 2-HIS mediating the migration of aryl moiety of LIG, are involved in the biosynthesis of DEIN (Fig. 3a). While the activity of AtC4H has been enhanced by co-expressing RP^77^ in our screening strains providing excess precursor *p*-HCA (QL11 background), the selected Ge2-HIS still exhibited sub-optimal performance in converting LIG to DEIN (Supplementary Fig. 4). Starting with evaluating plant CPRs and artificial RP surrogates, which could impact the transfer of electrons required for P450 activity, we therefore proceeded with the optimization of Ge2-HIS activity by exploring other endogenous metabolic factors, including heme metabolism, ER homeostasis, and NADPH generation. These modifications increased DEIN titer to a level exceeding 12 mg L^−1^ (Fig. 4b), accounting for a seven-fold improvement compared with the parental strain C33.

Another challenge for isoflavonoid production lies in overcoming the intrinsically low catalytic efficiency and/or selectivity of enzymes participating in the biosynthesis of plant secondary metabolites^78^. Gene amplification, by for example promoter engineering, is one approach to enhance enzyme activity. Here, implementation of dynamic expression control using inducible *GALps*, which enable a higher level of gene transcription than constitutive promoters^79^, boosted LIG production to 37.6 mg L^−1^ (Fig. 5b), a 284% increase relative to strain C09 having constitutive expression of the pathway genes. Spatial micro-compartmentalization via the formation of metabolons, which are ordered complexes of enzymes participating in sequential biosynthetic pathways, allows the effective formation of specialized metabolites and has shown to reduce metabolic crosstalk in plants^80^. To advance DEIN titers further, we therefore mimicked this natural phenomenon by bringing enzymes into proximity, using a linker-based fusion enzyme strategy, in turn greatly improving the metabolic flux through the LIG pathway and increasing its titer by 107% (Fig. 5b). Besides the AAA-derived *p*-HCA, de novo isoflavonoid biosynthesis consumes malonyl-CoA, whose formation is predominately invested in FAs synthesis in *S. cerevisiae*^61^. By fine-tuning the expression of key enzymes involved in FAs synthesis, we were able to redistribute the cellular malonyl-CoA pool, resulting in a 20% further increase in DEIN titer (Fig. 6f).

In conclusion, as a proof-of-concept study, a final DEIN titer of 85.4 mg L^−1^ was achieved using glucose as the sole carbon source in shake flask cultivations (Fig. 6g). This production level is comparable and, in some cases, higher than isoflavonoid levels produced by previous studies, which have additionally been aided with precursor feeding (Supplementary Table 2). Via further expression of different glycosyltransferases, approximately 80 mg L^−1^ of *C*- or *O*-glycosylated bioactive compounds PIN or DIN were produced (Fig. 7c), showing the application potential of our platform strain. Moreover, our work sheds light on the complete microbial biosynthesis of value-added isoflavonoids such as DEIN-derived legume phytoalexins^81^ and may be applied in characterizing novel metabolic enzymes for the production of isoflavonoid derivatives. Additional improvements on the catalytic efficiency and specificity of key isoflavonoid biosynthetic enzymes through protein engineering^78^, directed pathway evolution facilitated by biosensor-mediated high-throughput screening as well as engineering of extracellular transport of isoflavonoids^82,83^, may further optimize the phenotypes of our platform strains, including higher titer/productivity and reduction/elimination of byproducts, to meet industrial-scale production requirements in the future. Finally, the multi-faceted framework we herein present also offers the potential to be applied for engineering the biosynthetic pathways in other microbial hosts as well, for the production of complex natural products.

## Methods

### Strains, plasmids, and reagents

*Escherichia coli* DH5α strain was used for the construction and amplification of plasmids. All plasmids and *S. cerevisiae* strains used in this study are listed in Supplementary Table 3 and Supplementary Data 1, respectively. SapphireAmp® Fast PCR Master Mix and PrimeStar DNA polymerase were purchased from TaKaRa Bio. High-fidelity Phusion DNA polymerase and Gibson assembly kit were purchased from New England Biolabs. Plasmid miniprep, and DNA gel purification kits, and restriction enzymes were purchased from ThermoFisher Scientific. All codon-optimized plant and bacterial genes were chemically synthesized by GenScript and are listed in Supplementary Data 2. All primers (Supplementary Data 3) and chemicals (including analytical standards) were purchased from Sigma-Aldrich.

### Strain cultivation

YPD medium, consisting of 20 g L^−1^ peptone (Difco), 10 g L^−1^ yeast extract (Merck Millipore), and 30 g L^−1^ glucose (VWR), was used for routine yeast cultivation and preparation of competent cells. Synthetic complete medium without uracil (SC-URA), consisting of 6.7 g L^−1^ yeast nitrogen base (YNB) without amino acids (Formedium), 0.77 g L^−1^ complete supplement mixture without uracil (CSM-URA, Formedium), 20 g L^-1^ glucose (VWR) and 20 g L^−1^ agar (Merck Millipore), was used for selection of yeast transformants harboring the *URA3* marker. To drop out the *URA3* marker, yeast cultures were selected against on SC with 5-fluoroorotic acid (SC + 5-FOA) plates, containing 6.7 g L^−1^ YNB, 0.77 g L^−1^ complete supplement mixture and 0.8 g L^−1^ 5-FOA.

Shake flask batch fermentations for the production of isoflavonoid compounds were carried out in a defined minimal medium ((7.5 g L^−1^ (NH_4_)_2_SO_4_, 14.4 g L^−1^ KH_2_PO_4_, 0.5 g L^−1^ MgSO_4_ ∙ 7H_2_O, pH 6.0), 30 g L^−1^ glucose, 2 mL L^−1^ trace metal (3.0 g L^−1^ FeSO_4_ ∙ 7H_2_O, 4.5 g L^−1^ ZnSO_4_ ∙ 7H_2_O, 4.5 g L^−1^ CaCl_2_ ∙ 2H_2_O, 0.84 g L^−1^ MnCl_2_ ∙ 2H_2_O, 0.3 g L^−1^ CoCl_2_ ∙ 6H_2_O, 0.3 g L^−1^ CuSO_4_ ∙ 5H_2_O, 0.4 g L^−1^ Na_2_MoO_4_ ∙ 2H_2_O, 1.0 g L^−1^ H_3_BO_3_, 0.1 g L^−1^ KI and 19.0 g L^−1^ Na_2_EDTA ∙ 2H_2_0) and 1 mL L^−1^ vitamin solutions (0.05 g L^−1^ D-Biotin, 1.0 g L^−1^ D-Pantothenic acid hemicalcium salt, 1.0 g L^−1^ Thiamin-HCl, 1.0 g L^−1^ Pyridoxin-HCl, 1.0 g L^−1^ Nicotinic acid, 0.2 g L^−1^ 4-aminobenzoic acid, 25.0 g L^−1^ *myo*-Inositol)^84^ supplemented with 60 mg L^−1^ uracil and 1 mM 5-aminolevulinic acid (5-ALA) if required. Single colonies, with PCR-verified genetic modifications, were inoculated into 14 mL tubes with 1.5 mL fresh minimal medium and incubated at 30 °C with 220 rpm agitation overnight. Precultures were then diluted and transferred to a 125 mL non-baffled flask containing 15 mL minimal medium at an initial optical density measured at 600 nm (OD600) of 0.05 and cultivated at 220 rpm, 30 °C for an additional 72 h. For mimicked fed-batch shake flask cultivations, six tablets of FeedBeads^60^ (SMFB08001, Kuhner Shaker, Basel, Switzerland) were used as the sole carbon source and cultivated for 90 h at 30 °C with 220 rpm agitation (corresponding to 30 g L^−1^ glucose). To induce the transcription of genes under the control of *GALps*, 10 g L^−1^ galactose was added into the medium as the gratuitous inducer.

Analysis of the growth-inhibitory effect of DEIN and its glucosides on yeast was performed using Growth Profiler 960 (EnzyScreen). Overnight precultures were diluted and transferred to 96-well microplates containing 250 µL defined minimal medium with the initial OD600 of 0.1. DEIN was added to the wells in final concentrations of 25, 100 and 150 mg L^−1^. PIN was added in a final concentration of 50, 500, 1000 and 2000 mg L^−1^. DIN was added in a final concentration of 25, 50 and 250 mg L^−1^. The cultures were grown at 30 °C with 250 rpm shaking, and the OD600 was measured with an interval of 30 min.

### Genetic manipulation

All constructed yeast strains were derived from the genetic background *S. cerevisiae* CEN.PK113-5D-derivative IMX581 (*MATa ura3-52 can1∆::cas9-natNT2 TRP1 LEU2 HIS3*)^85^. For gene overexpression, in-vitro-assembled DNA constructs were integrated at target genomic loci by means of the *CRISPR/cas9* system. For the amplification of native promoters, genes, and terminators, IMX581 genomic DNA served as the template. Plasmids or synthetic fragments were utilized as the DNA template to amplify optimized heterologous genes (Supplementary Table 3 and Supplementary Data 2). High-fidelity Phusion DNA polymerase was used for routine DNA fragment amplifications, except PrimeSTAR HS polymerase was selected for in vitro fusion PCR for generating integration constructs. Functional expression modules were generated according to an overlapping extension PCR (OE-PCR) procedure^86^. All used integration cassettes are listed in Supplementary Data 4.

Specific chromosomal loci, enabling stable and high-level expression of heterologous genes^87^, were selected as the recombination sites for integration of gene overexpression constructs. Construction of strain C09 was used as an example to show the *CRISPR/cas9*-mediated integration of in-vitro-assembled DNA constructs. Fragment M1 was generated through assembling DNA parts *XII-1 us*, *CYC1t*, *At4CL1*, *TDH3p*, *CCW12p*, *GmCHR5*, *FBA1t* and *TPS1t*. In detail, the upstream homologous arm *XII-1 us* (P190/P191), terminators *CYC1t* (P25/P26), *FBA1t* (P27/P29) and *TPS1t* (P30/P31), and promoters *TDH3p* (P1/P2) and *CCW12p* (P4/P5) were amplified from IMX581 genomic DNA, respectively; and coding regions of *At4CL1* (P44/P48) and *GmCHR5* (P62/P66) were amplified using pCfB0854 and synthetic gene as the template, respectively. Fragment M14 was assembled by fusing the DNA parts of *FBA1t*, *TPS1t*, *GmCHS8*, *TEF1p*, *PGK1p*, *GmCHI1B2*, *ADH1t*, and *XII-1 ds*. Specifically, coding regions of *GmCHS8* (P85/P89) and *GmCHI1B2* (P100/P104) were amplified from synthetic genes; promoters *TEF1p* (P6/P7) and *PGK1p* (P8/P9), terminator *ADH1t* (P32/P33) and the downstream homologous arm *XII-1 ds* (P192/P193) were amplified from IMX581 genomic DNA, respectively. Subsequently, equimolar amounts of purified fragments M1 and M14 (100 ng kb^−1^) with *XII-1* targeting gRNA vector pQC032 (~300 ng) were mixed and co-transformed into *p*-HCA-producing strain QL11 using the lithium acetate-mediated yeast transformation protocol^85^. The resulting transformants were selected on SC-URA plates and colony PCR using SapphireAmp® Fast PCR Master Mix was performed to identify correct integrants. For gene deletion, 2 µg of a double-stranded DNA fragment consisting of two 50 bp sequences homologous to the flanking sequences of target genes, serving as the homologous repair of the genome double-strand break introduced by the cleavage of Cas9 nuclease, were co-transformed with corresponding gRNA vectors. Likewise, resulting transformants were selected on SC-URA plates and colony PCR using SapphireAmp® Fast PCR Master Mix was performed to identify correct deletants. To construct the fusion protein of adjacent metabolic enzymes, two types of flexible linker GGGS (flexible) and VDEAAAKSGR (rigid) were evaluated. For the construction of the *FAS1* promoter-substitution strains, the native promoter sequences of *FAS1* (from −90 to 0 bp) were replaced by selected promoters in Supplementary Table 1 using the *CRISPR/cas9* system. Galactose-degrading genes *GAL7/10/1* were deleted to enable galactose as a gratuitous inducer for the transcription of genes under the control of *GAL* promoters. A schematic overview of all strain construction is shown in Supplementary Fig. 2.

To obtain specific guide RNAs for a selected gene/genomic locus, all potential gRNAs were identified and ranked with CEN.PK113-7D genetic background using the free and open CRISPRdirect tool (http://crispr.dbcls.jp/)^88^. All single and double gRNA plasmids were constructed according to the Gibson assembly method in which gRNA sequence-containing DNA parts were in vitro recombined with a vector backbone^85^. Correct recombinant plasmids were then verified by sequencing. To construct bidirectional promoter vector pQC223, a fragment consisting of *GAL1p-GAL10p* (P16/P24) was amplified from IMX581 genomic DNA, gel-purified and recombined with a *Kpn* I/*Sac* I-digested pSP-GM1 backbone using Gibson assembly method. *E. coli* colony PCR was then performed to identify correct recombinant plasmids which were further confirmed by sequencing.

### Metabolite extraction and quantification

Isoflavonoids and aromatic metabolite production were quantified by high-performance liquid chromatography (HPLC)^27^. In detail, 0.5 mL of cell culture was mixed with an equal volume of absolute ethanol (100% v/v), vortexed thoroughly and centrifuged at 13,000 × *g* for 5 min. The supernatant was stored at −20 °C until HPLC analysis. Quantification of isoflavonoids and aromatics was performed on a Dionex Ultimate 3000 HPLC (ThermoFisher Scientific, Waltham, MA, USA) equipped with a Discovery HS F5 15 cm × 4.6 mm column (particle size 5 µm, Sigma-Aldrich, St. Louis, MO, USA) connected to a photodiode array (PDA) detector (250, 270, 290, 304 and 370 nm). The column was kept at 30 °C, and metabolites from 10 µL of supernatants were separated. Samples were analyzed using a gradient method with two solvents: water with 0.1% formic acid (A) and acetonitrile (B). For *p*-HCA, NAG, GEIN, ISOLIG, LIG, DEIN, DIN, PIN, GIN, and G8G detection, a flow rate of 1.2 ml min^−1^ was used. The program started with 5% of solvent B (0–0.5 min), after which its fraction was increased linearly from 5 to 60% (0.5–18.5 min), then the fraction was maintained at 60% (18.5–19 min), after that the fraction was decreased from 60 to 5% (19–19.5 min), finally, the fraction was maintained at 5% (19.5–20 min). *p*-HCA was detected at 9.3 min (304 nm), NAG at 14.8 min (290 nm), GEIN at 14.5 min (270 nm), ISOLIG at 16.3 min (370 nm), LIG at 12.8 min (270 nm), DEIN at 12.0 min (250 nm), DIN at 8.1 min (250 nm), PIN at 7.1 min (250 nm), GIN at 9.7 min (250 nm) and G8G at 8.7 min (250 nm). Chromeleon was used for HPLC data collection. Compound identity was confirmed by comparing the UV absorbance spectra and retention times of the samples with authentic standards. A six-point calibration curve, ranging from 6.25 mg L^−1^ to 200 mg L^−1^ (*p*-HCA), 3.125 mg L^−1^ to 100 mg L^−1^ (NAG), and 1.5625 mg L^−1^ to 50 mg L^−1^ (GEIN, ISOLIG, LIG, DEIN, DIN, PIN, GIN and G8G), was generated for the quantification of these chemicals. The *R*^2^ coefficient for the resulting calibration curve was 0.99. Quantitative analysis was carried out using Microsoft Excel.

The glucose release kinetic of the FeedBeads was determined in a minimal medium without a carbon source. Briefly, six tablets of FeedBeads were placed in a 125 mL non-baffled flask containing 15 mL minimal medium and incubated at 30 °C with an agitation rate of 220 rpm. 50 µL cultures were removed from the flask at multiple time points and centrifuged at 13,000 × *g* for 5 min. The supernatant was then stored at −20 °C until further analysis. The concentration of glucose was quantified by HPLC analysis on an Aminex HPX-87G column (Bio-Rad) on an Ultimate 3000 HPLC with a refractive index detector. The column was eluted with 5 mM H_2_SO_4_ at a flow rate of 0.6 mL min^−1^ at 45 °C for 35 min. Chromeleon was used for HPLC data collection and Microsoft Excel for further quantitative analysis.

### Identification of glycosylated products

Liquid chromatography-mass spectrometry (LC-MS) analysis was performed to verify the production of PIN and DIN by engineered yeast cells. Specifically, strains C28, E03, and E06 were cultivated in 15 mL minimal medium with 30 g L^−1^ glucose for 72 h. For the LC-MS sample preparation, 2 mL resultant cell culture was collected and freeze-dried in a Christ Alpha 2-4LSC for 48 h. Then, 1 mL of absolute ethanol was added, vigorously vortexed for 10 min, and centrifuged at 13,000 × *g* for 5 min. The supernatant was collected, fully dried under vacuum, and resuspended with 200 μL absolute ethanol.

Ten microliters of each sample was injected and analyzed on an Agilent Infinity 1290 UHPLC connected to an Agilent 6520 high-resolution mass spectrometry. The UHPLC used a Waters UPLC HSS T3 10 cm × 2.1 mm column (particle size 1.8 µm). The column temperature was set to 45 °C and the flow rate was 0.4 ml min^−1^ with a solvent system containing 0.04% formic acid (solvent A) and methanol with 0.04% formic acid (solvent B). The gradient started at 5% solvent B and ramped to 100% solvent B over 6 min and held for 4.5 min. The LC eluent was directed to the MS equipped with a Dual electrospray ionization (ESI) source in a positive ionization mode scanning from 50 to 1200 m/z at 1.67 spectra s^−1^. The capillary voltage was set at 3500 V. The source parameters were set with a gas temperature at 175 °C, gas flow at 10 L min^−1^, and nebulizer at 45 psig. MS data were acquired with Agilent MassHunter LC-MS Data Acquisition and analyzed using Masshunter Qualitative analysis.

### Reporting summary

Further information on research design is available in the Nature Research Reporting Summary linked to this article.

## Supplementary information

Supplementary Information

Description of Additional Supplementary Files

Supplementary Data 1

Supplementary Data 2

Supplementary Data 3

Supplementary Data 4

Reporting Summary

## Data Availability

Data supporting the findings of this study are available within the article and its Supplementary Information files. The GenBank (https://www.ncbi.nlm.nih.gov/genbank/) accession numbers and codon-optimized nucleotide sequences of the genes referenced in this study are provided in this paper. All other data that support the findings of this study are available from the corresponding author upon request. All plasmids and strains used in this study are available from the corresponding author under a material transfer agreement. Source data are provided with this paper.

## Source data

Source Data
