# Supplementary material for: De novo biosynthesis of bioactive isoflavonoids by engineered yeast cell factories
Source: Nat Commun. 2021 Oct 19;12:6085. doi: 10.1038/s41467-021-26361-1 (PMC8526750; doi:10.1038/s41467-021-26361-1)
Supplement: Supplementary file 1 — Supplementary Information [file 41467_2021_26361_MOESM1_ESM.pdf]

Supplementary Information for

De novo biosynthesis of bioactive isoflavonoids by engineered yeast cell factories

Quanli Liu[1,2#], Yi Liu[1,2#], Gang Li[1,2], Otto Savolainen[1,3,4], Yun Chen[1,2], Jens Nielsen[1,2,5,6*]

[1]Department of Biology and Biological Engineering, Kemivägen 10, Chalmers University of Technology, SE41296 Gothenburg, Sweden

[2]Novo Nordisk Foundation Center for Biosustainability, Chalmers University of Technology, SE41296 Gothenburg, Sweden

[3]Chalmers Mass Spectrometry Infrastructure, Kemivägen 10, Chalmers University of Technology, SE41296 Gothenburg, Sweden

[4]Institute of Public Health and Clinical Nutrition, University of Eastern Finland, FI70211 Kuopio, Finland

[5]Novo Nordisk Foundation Center for Biosustainability, Technical University of Denmark, DK2800 Kongens Lyngby, Denmark

[6]BioInnovation Institute, Ole Maaløes vej 3, DK2200 Copenhagen N, Denmark.

[#]These authors contributed equally: Quanli Liu, Yi Liu.

[*] Corresponding author E-mail: nielsenj@chalmers.se

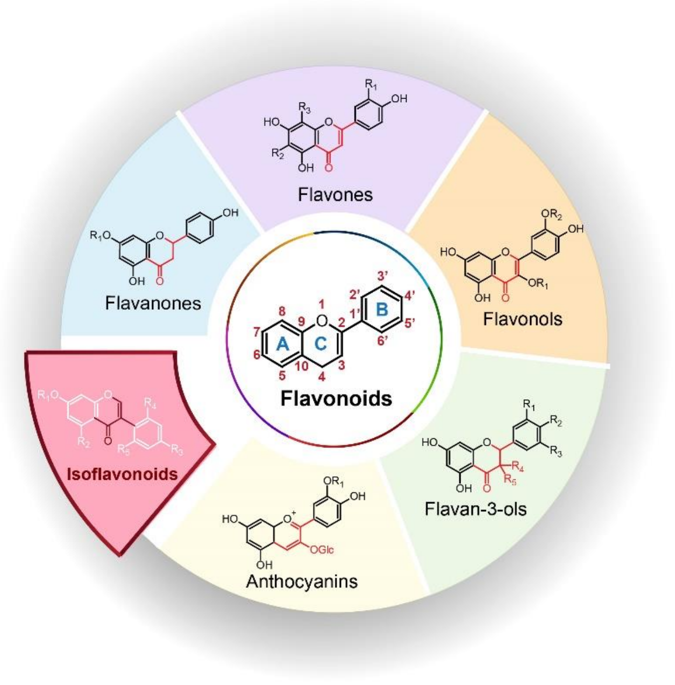

Supplementary Fig. 1. Main subfamilies and core skeletons of common flavonoids. Isoflavonoids contain the common C6-C3-C6 flavonoid skeleton and are characterized by having the B-ring connected at C3 rather than C2 position of the ring C, compared to other flavonoid subclasses

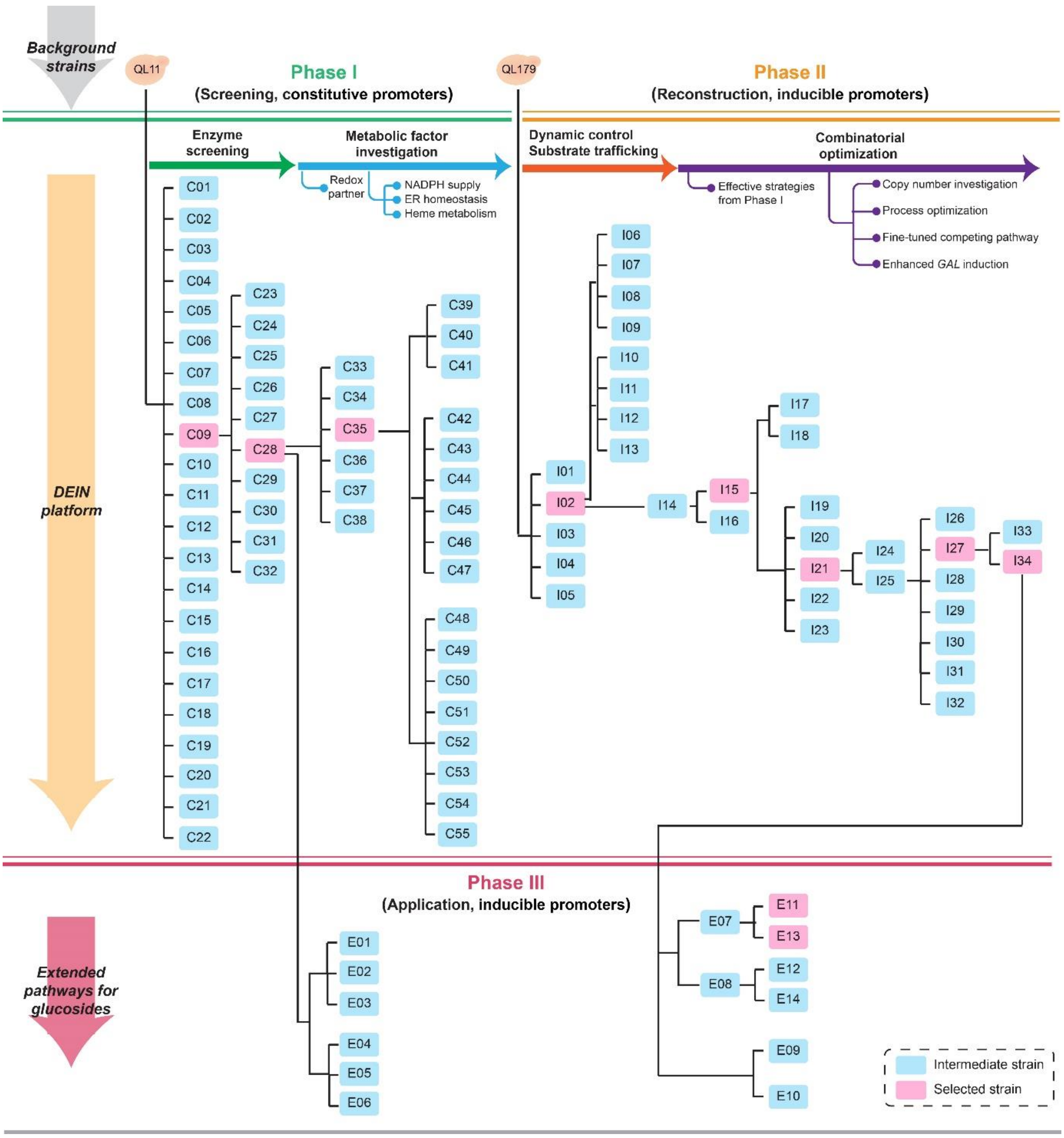

Supplementary Fig. 2. Flowchart of yeast strain construction in this study.

a

4CL	---	At4CL1	At4CL1	At4CL1	At4CL1	At4CL1	At4CL1	At4CL1	At4CL1	At4CL1	At4CL1	At4CL1
**CHR**	---	GmCHR5	GmCHR5	GmCHR5	PlCHR	PlCHR	MsCHR	MsCHR	GmCHR5	GmCHR5	PlCHR	MsCHR
**CHS**	---	RsCHS	RsCHS	RsCHS	RsCHS	RsCHS	RsCHS	RsCHS	PlCHS	GmCHS8	PlCHS	PlCHS
**CHI**	---	PsCHI	PlCHI1	GmCHI1B2	PlCHI1	GmCHI1B2	PlCHI1	GmCHI1B2	GmCHI1B2	GmCHI1B2	GmCHI1B2	GmCHI1B2
**Strain**	QL11	C01	C02	C03	C04	C05	C06	C07	C08	C09	C10	C11
**4CL**		Pl4CL1	At4CL1m	Gm4CL3	At4CL2	Ph4CL	Pc4CL2	At4CL1	At4CL1	At4CL1	At4CL1	At4CL1
**CHR**		GmCHR5	GmCHR5	GmCHR5	GmCHR5	GmCHR5	GmCHR5	GmCHR5	GmCHR5	GmCHR5	GmCHR5	GmCHR5
**CHS**		GmCHS8	GmCHS8	GmCHS8	GmCHS8	GmCHS8	GmCHS8	HaCHS	PhCHS	SbCHS2	GmCHS8	GmCHS8
**CHI**		GmCHI1B2	GmCHI1B2	GmCHI1B2	GmCHI1B2	GmCHI1B2	GmCHI1B2	GmCHI1B2	GmCHI1B2	GmCHI1B2	MsCHI	PlCHI1
**Strain**		C12	C13	C14	C15	C16	C17	C18	C19	C20	C21	C22

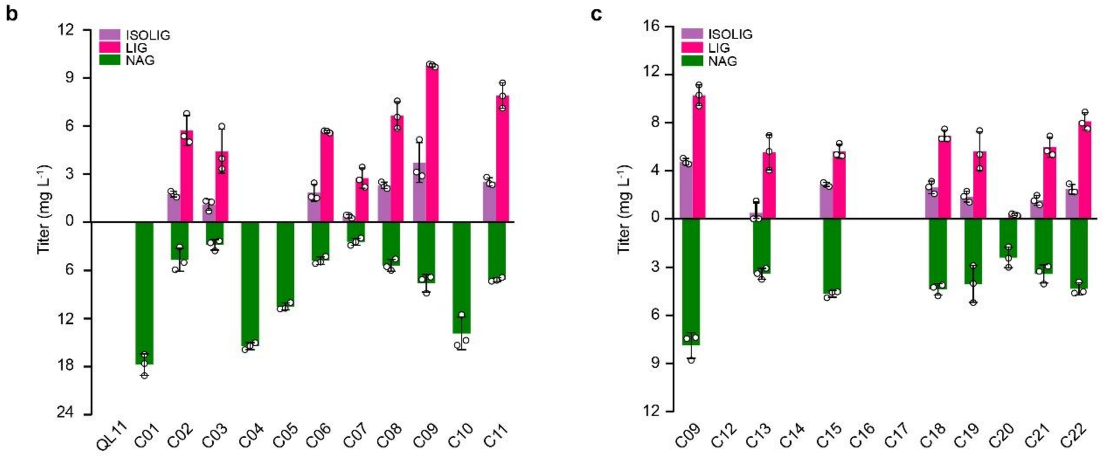

Supplementary Fig. 3. Characterization and comparison of metabolic enzymes for production of LIG. **a** Genetic characteristics of generated yeast strains. For the source of selected plant genes: *Ms*, *Medicago sativa*; *Rs*, *Rhododendron simsii*; *Ha*, *Hypericum androsaemum*; *Ph*, *Petunia hybrida*; *Sb*, *Scutellaria baicalensis*; *Ps*, *Paeonia suffruticosa*. See Fig. 1 and 2 legends regarding gene details and abbreviations of other plant species. **b**-**c** Production profiles of intermediates ISOLIG and LIG and by-product NAG produced by yeast strains harboring different combination of biosynthetic enzymes 4CL, CHS, CHR and CHI. Cells were grown in defined minimal medium with 30 g L^{-1} glucose as the sole carbon source, and cultures were sampled after 72 h of growth for metabolite detection. All data represent the mean of n = 3 biologically independent samples and error bars show standard deviation. The source data underlying figures **b**-**c** are provided in a Source Data file.

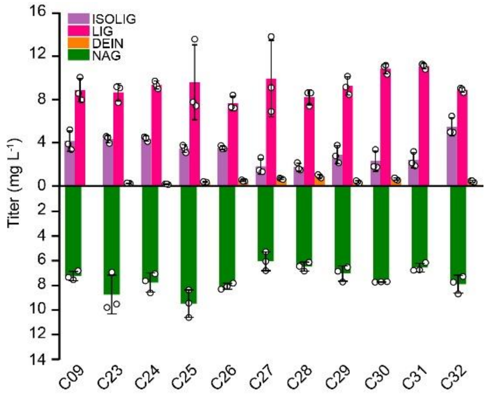

Supplementary Fig. 4. Production profiles of yeast strains overexpressing different biosynthetic genes encoding 2-HIS and HID. Titers of DEIN, its intermediates and by-products are shown. Cells were grown in defined minimal medium with 30 g L^{-1} glucose as the sole carbon source, and cultures were sampled after 72 h of growth for metabolite detection. All data represent the mean of n = 3 biologically independent samples and error bars show standard deviation. The source data are provided in a Source Data file.

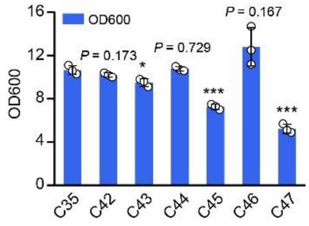

Supplementary Fig. 5. Growth profiles of strains generated by engineering ER homeostasis. Cells were grown in defined minimal medium with 30 g L^{-1} glucose as the sole carbon source, and cultures were sampled after 72 h of growth for OD600 measurement. Statistical analysis was performed by using Student's *t* test (two-tailed; two-sample unequal variance; *$p < 0.05$, **$p < 0.01$, ***$p < 0.001$). All data represent the mean of n = 3 biologically independent samples and error bars show standard deviation. The source data are provided in a Source Data file.

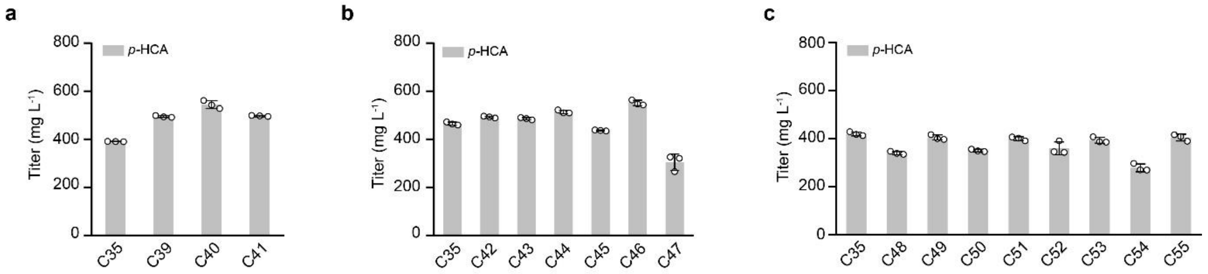

Supplementary Fig. 6. Residual precursor *p*-HCA of engineered yeast strains. The *p*-HCA profiles of strains generated by engineering heme metabolism (**a**), ER homeostasis (**b**) and the cofactor NADPH generation (**c**) were measured. See Fig. 4 for more strain and gene details. Cells were grown in defined minimal medium with 30 g L^{-1} glucose as the sole carbon source, and cultures were sampled after 72 h of growth for metabolite detection. All data represent the mean of n = 3 biologically independent samples and error bars show standard deviation. The source data are provided in a Source Data file.

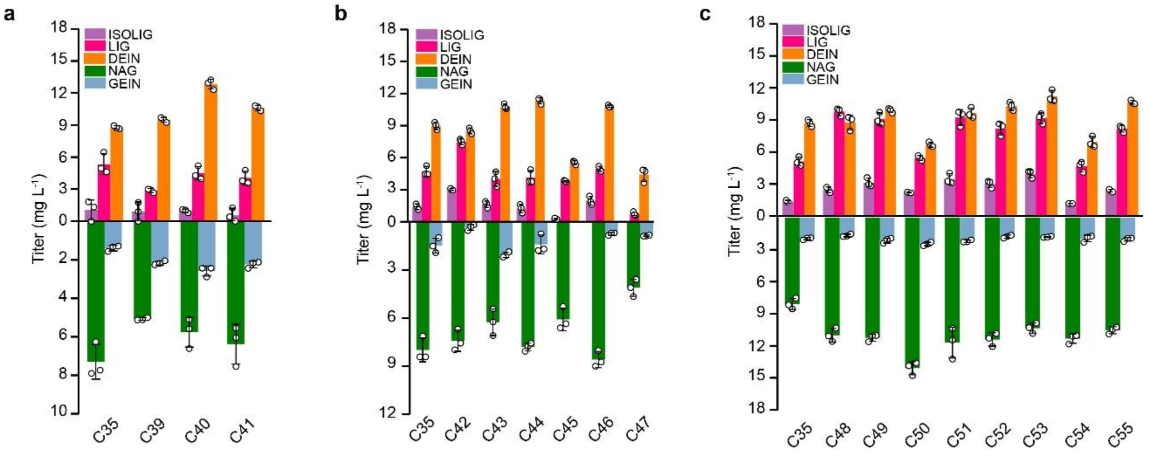

Supplementary Fig. 7. Production profiles of engineered yeast strains. Titers of DEIN, its intermediates and by-products of strains generated by engineering heme metabolism (**a**), ER homeostasis (**b**) and cofactor NADPH generation (**c**) are shown. See Fig. 4 for more strain and gene details. Cells were grown in defined minimal medium with 30 g L^{-1} glucose as the sole carbon source, and cultures were sampled after 72 h of growth for metabolite detection. All data represent the mean of n = 3 biologically independent samples and error bars show standard deviation. The source data are provided in a Source Data file.

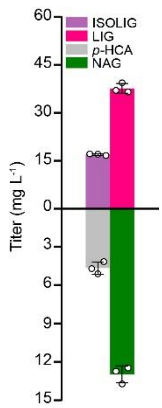

Supplementary Fig. 8. Metabolic intermediates and by-product produced by strains I01 expressing DEIN pathway under the control of inducible *GAL* promoters. Cells were grown in defined minimal medium with 30 g L^{-1} glucose as the sole carbon source and 10 g L^{-1} galactose as the inducer. Cultures were sampled after 72 h of growth for metabolite detection. All data represent the mean of n = 3 biologically independent samples and error bars show standard deviation. The source data are provided in a Source Data file.

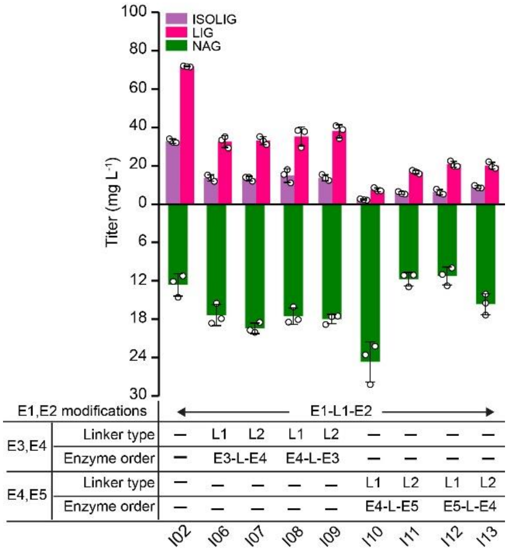

Supplementary Fig. 9. Metabolic intermediates and by-product produced by strains expressing fused enzymes E3 (GmCHS8)-Linker-E4 (GmCHR5) and E4 (GmCHR5)-Linker-E5(GmCHI1B2). Cells were grown in defined minimal medium with 30 g L^{-1} glucose as the sole carbon source and 10 g L^{-1} galactose as the inducer. Cultures were sampled after 72 h of growth for metabolite detection. All data represent the mean of n = 3 biologically independent samples and error bars show standard deviation. The source data are provided in a Source Data file.

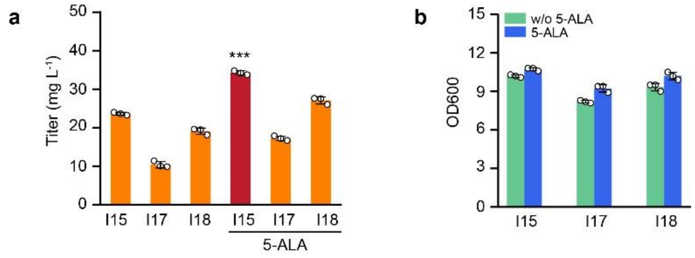

Supplementary Fig. 10. Effect of 5-ALA supplementation on DEIN production and cell growth. The production of DEIN (**a**) and optical density at 600nm (**b**) were measured. Cells were grown in defined minimal medium with 30 g L^{-1} glucose as the sole carbon source and 10 g L^{-1} galactose as the inducer. Cultures were were sampled after 72 h of growth for metabolite detection. Statistical analysis was performed by using Student's *t* test (two-tailed; two-sample unequal variance; *$p < 0.05$, **$p < 0.01$, ***$p < 0.001$). All data represent the mean of n = 3 biologically independent samples and error bars show standard deviation. The source data are provided in a Source Data file.

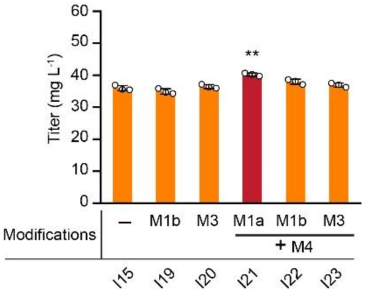

Supplementary Fig. 11. DEIN production of strains integrated with strategies altering NADPH generation. See Fig. 4 for more strain and gene information. Cells were grown in defined minimal medium with 30 g L^{-1} glucose as the sole carbon source and 10 g L^{-1} galactose as the inducer. Cultures were sampled after 72 h of growth for metabolite detection. Statistical analysis was performed by using Student's *t* test (two-tailed; two-sample unequal variance; *$p < 0.05$, **$p < 0.01$, ***$p < 0.001$). All data represent the mean of n = 3 biologically independent samples and error bars show standard deviation. The source data are provided in a Source Data file.

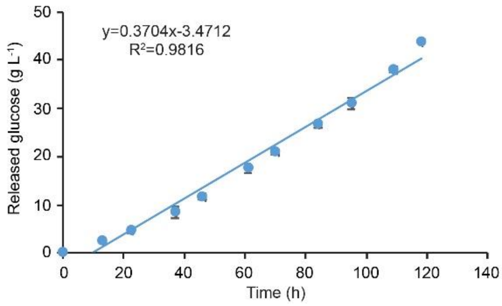

Supplementary Fig. 12. Glucose release profile of FeedBeads in defined minimal medium. Six tablets of FeedBeads (FB), a slow release system for glucose, were placed in a 125 ml non-baffled flask containing 15 ml minimal medium and incubated at 30 °C with an agitation rate of 220 rpm. 50 µl cultures were removed from the flask at indicated time points for quantification of glucose concentration. All data represent the mean of n = 3 biologically independent samples and error bars show standard deviation. The source data are provided in a Source Data file.

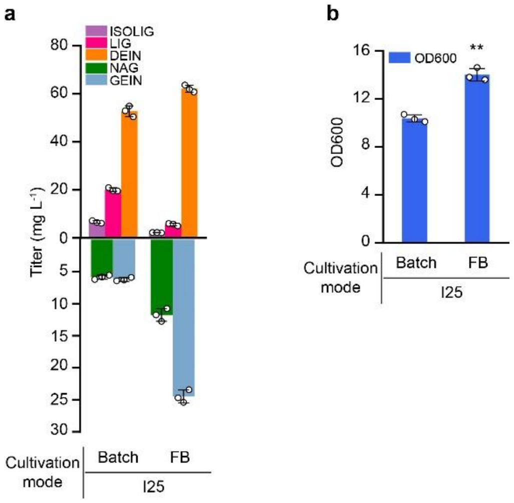

Supplementary Fig. 13. Phenotypes of strain I25 under cultivations with different modes of carbon source supply. See Fig. 6 for more strain details. The production of DEIN, intermediates and by-products (**a**) and optical density at 600nm (**b**) were measured. Cells were grown in defined minimal medium with 30 g L^{-1} glucose (Batch) or six tablets of FB as the sole carbon source and 10 g L^{-1} galactose as the inducer. Cultures were sampled after 72 h (Batch) or 90 h (FB) of growth for metabolite detection. Statistical analysis was performed by using Student's *t* test (two-tailed; two-sample unequal variance; *p < 0.05, **p < 0.01, ***p < 0.001). All data represent the mean of n = 3 biologically independent samples and error bars show standard deviation. The source data are provided in a Source Data file.

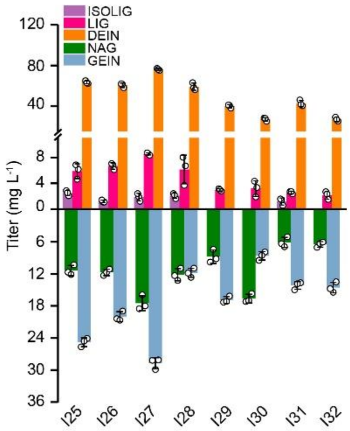

Supplementary Fig. 14. Production profiles of strains harboring regulated expression of *FAS1* gene. The production of DEIN, intermediates and by-products were measured. Cells were grown in defined minimal medium with six tablets of FB as the sole carbon source and 10 g L^{-1} galactose as the inducer. Cultures were sampled after 90 h of growth for metabolite detection. All data represent the mean of n = 3 biologically independent samples and error bars show standard deviation. The source data are provided in a Source Data file.

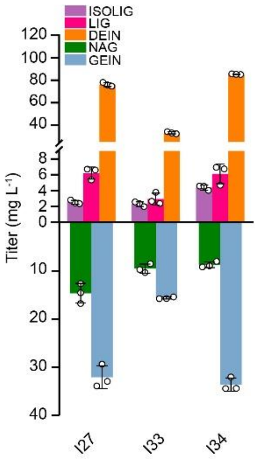

Supplementary Fig. 15. Production profiles of strains integrated with strategies regulating the *GAL* induction. The production of DEIN, intermediates and by-products were measured. Cells were grown in defined minimal medium with six tablets of FB as the sole carbon source and 10 g L^{-1} galactose as the inducer. Cultures were sampled after 90 h of growth for metabolite detection. All data represent the mean of n = 3 biologically independent samples and error bars show standard deviation. The source data are provided in a Source Data file.

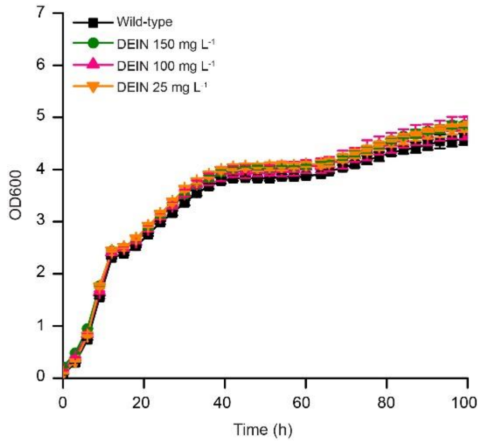

Supplementary Fig. 16. Growth profiles of *S. cerevisiae* under different concentrations of DEIN. Overnight precultures of background strain IMX581 were diluted and transferred to 96-well microplates containing 250 µl defined minimal medium with the initial OD600 of 0.1. The cultures were grown at 30°C with 250 rpm shaking, and the OD600 was automatically measured by Growth Profiler 960 (EnzyScreen). All data represent the mean of n = 3 biologically independent samples and error bars show standard deviation. The source data are provided in a Source Data file.

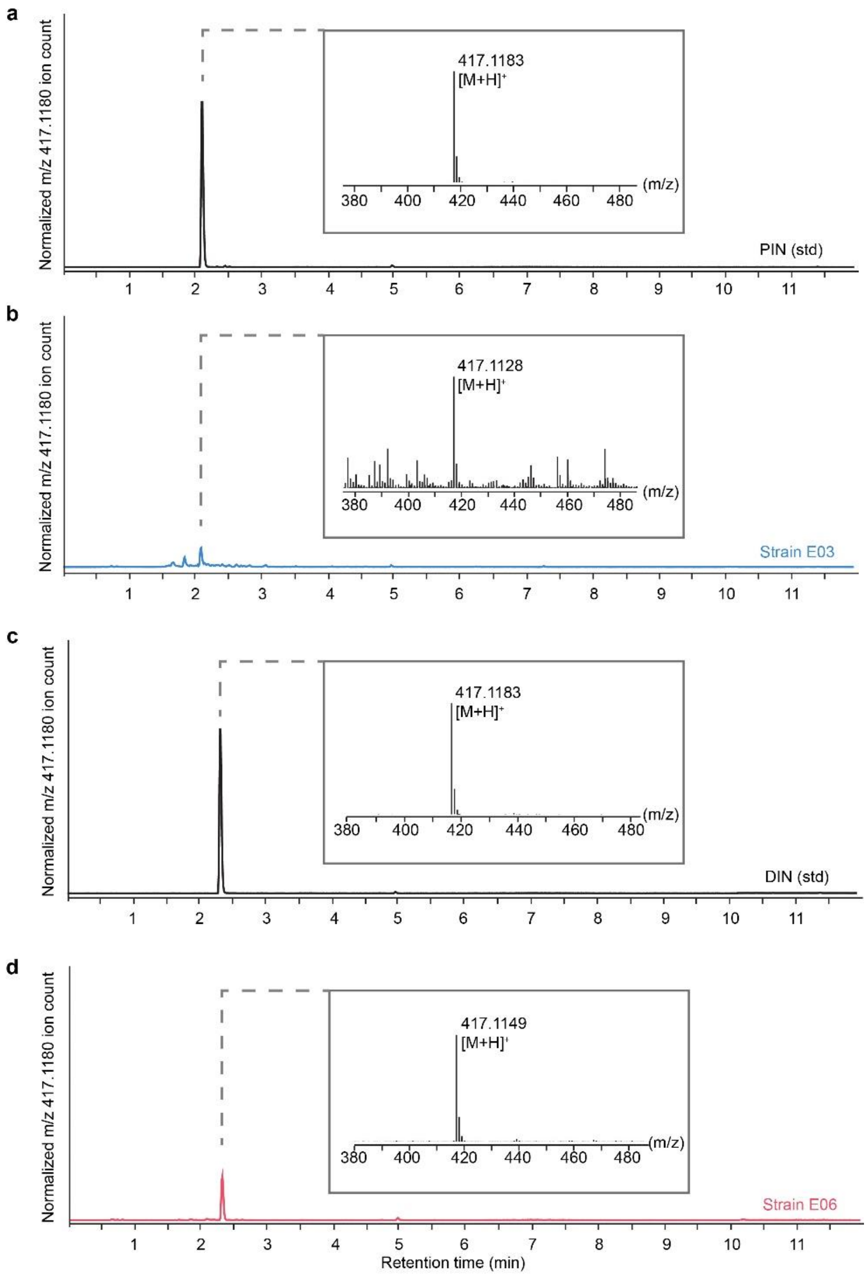

Supplementary Fig. 17. LC-MS characterization of glycosylated chemicals. Extracted ion chromatograms at m/z$^+$ 417.1180 are shown as evidence for the production of PIN and DIN in engineered yeast strains. LC-MS profiles are shown for the analysis of PIN standard (**a**), the PIN product from *PlUGT43*-expressing strain E03 extracts (**b**), DIN standard (**c**) and the DIN product from the *GmUGT4*-expressing strain E06 extracts (**d**). Cells were grown in defined minimal medium with 30 g L^{-1} glucose as the sole carbon source, and cultures were sampled after 72 h of growth for LC-MS analysis.

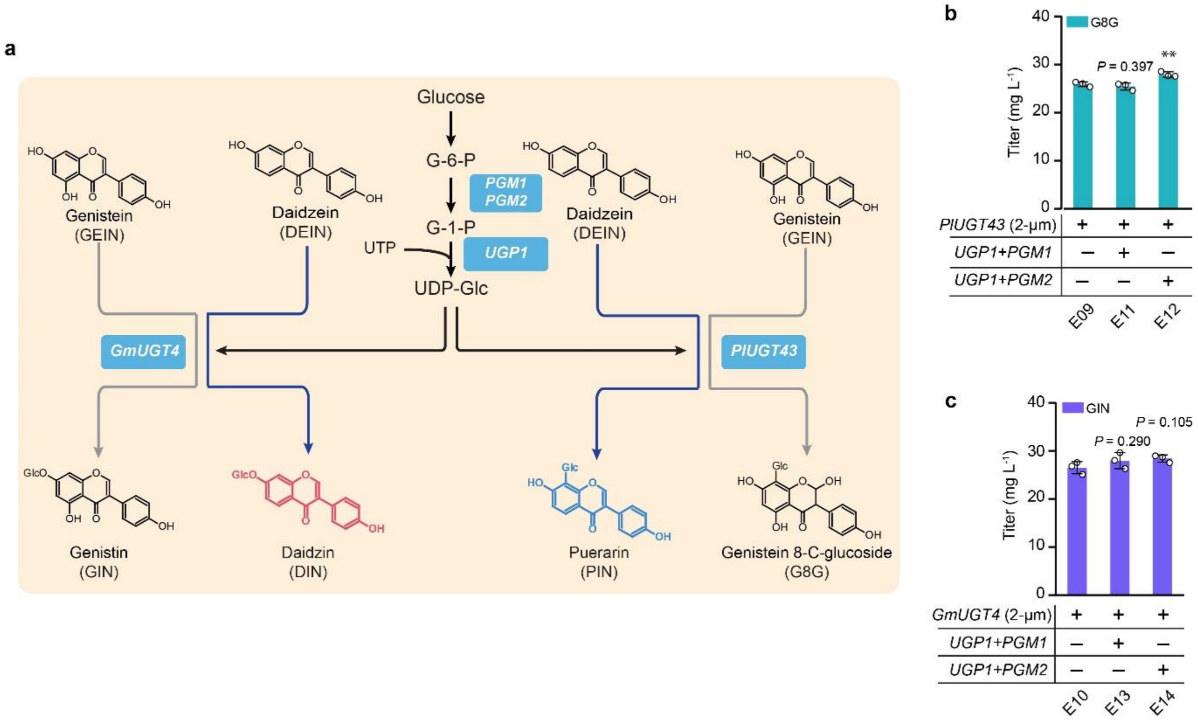

Supplementary Fig. 18. Production of glycosylated chemicals. a Schematic view of the engineered metabolic pathways for the biosynthesis of glucosides DIN and PIN, and relevant by-products. See Fig. 1 legend regarding abbreviations of metabolites and gene details. Production profiles of G8G (**b**) and GIN (**c**) in DEIN hyper-producing strain I34 background with or without increased UDP-glucose supply. Cells were grown in defined minimal medium with six tablets of FB as the sole carbon source and 10 g L^{-1} galactose as the inducer. Cultures were sampled after 90 h of growth for metabolite detection. All data represent the mean of n = 3 biologically independent samples and error bars show standard deviation. The source data underlying figures **b**-**c** are provided in a Source Data file.

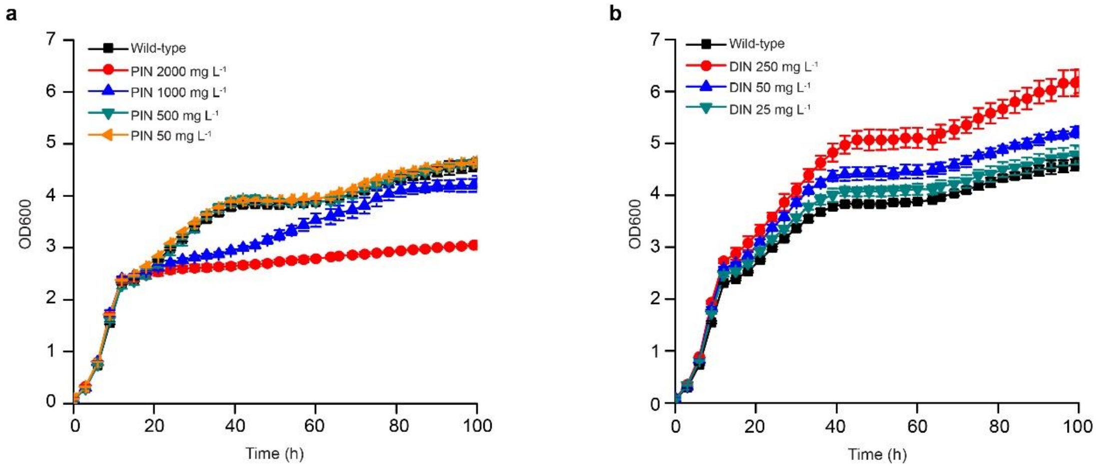

Supplementary Fig. 19. Toxicity for growth of *S. cerevisiae* of DEIN glucosides. Growth profiles of background strain IMX581 under different concentrations of PIN (**a**) and DIN (**b**) were evaluated. Overnight precultures were diluted and transferred to 96-well microplates containing 250 µl defined minimal medium with the initial OD600 of 0.1. The cultures were grown at 30°C with 250 rpm shaking, and the OD600 was automatically measured by Growth Profiler 960 (EnzyScreen). All data represent the mean of n = 3 biologically independent samples and error bars show standard deviation. The source data are provided in a Source Data file.

Supplementary Table 1. Promoters used for fine-tuning the transcription of *FAS1* gene in yeast

No.	Alias	Activity on Glucose[a]
Native	*FAS1p*	100%
1	*PFK2p*	87%
2	*BGL2p*	83%
3	*HXT2p*	72%
4	*QCR9p*	63%
5	*PRE2p*	61%
6	*TIF5p*	55%
7	*YAP1p*	25%

[a] The transcriptional activities of selected promoters are obtained from previous report[1].

Supplementary Table 2. Microbial production of isoflavonoids in shake flask cultivations.

Compound	Host	Biosynthetic profile	Precursor feeding	Titer (mg L^{-1})	Reference
GEIN	*E. coli/ S. cerevisiae*	Bioconversion	L-Tyrosine	6	[2]
	E. coli	Bioconversion	NAG	10 mg/g DCW[a]	[3]
	S. cerevisiae	Bioconversion	NAG	0.5 mg/g DCW	[3]
	E. coli	Bioconversion	NAG	16.2	[4]
	E. coli	Bioconversion	NAG	35	[5]
	S. cerevisiae	Bioconversion	NAG	7.7	[6]
	S. cerevisiae	Bioconversion	NAG	35	[7]
GIN	*E. coli*	Bioconversion	GEIN	20	[8]
	E. coli	Bioconversion	GEIN	60.5	[9]
DEIN	*E. coli*	Bioconversion	LIG	18 mg/g DCW	[3]
	S. cerevisiae	Bioconversion	LIG	2 mg/g DCW	[3]
	S. cerevisiae	*De novo*	None	85.4 (9 mg/g DCW)	This Study
DIN	*E. coli*	Bioconversion	DEIN	51.7	[9]
	S. cerevisiae	*De novo*	None	73.2	This Study
PIN	*S. cerevisiae*	*De novo*	None	72.8	This Study

[a] DCW, dry cell weight.

Supplementary Table 3. Plasmids used in this study.

Plasmid ID	Relevant characteristics	Reference
**Template plasmids**		
pCfB0854	Template for ***At4CL1***	[10]
pQC155	*pUC Ori ampR* template for ***At4CL1m (I250L, N404K, I461V)***	This study
pCfB0855	Template for ***At4CL2***	[10]
pQC221	*pUC Ori ampR* template for ***GAL3***	This study
pQC222	*pUC Ori ampR* template for ***GAL3***^{S509P}	This study
**Gene overexpression**		
pSP-GM1	2µm *ampR URA3 TEF1p-ADH1t*, *PGK1p-CYC1t*	[11]
pQC223	2µm *ampR URA3 GAL1p-ADH1t*, *GAL10p-CYC1t*	This study
pQC229	2µm *ampR URA3 GAL1p-**PlUGT43**-ADH1t*, *GAL10p-CYC1t*	This study
pQC230	2µm *ampR URA3 GAL1p-**GmUGT4**-ADH1t*, *GAL10p-CYC1t*	This study
pQC231	2µm *ampR URA3 GAL1p-ADH1t*, *GAL10p-**GAL3***^{S509P}*-CYC1t*	This study
**gRNA vectors**		
pMEL10	2µm *ampR KlURA3* gRNA-*CAN1*.Y	[12]
pROS10	2µm *ampR URA3* gRNA-*CAN1*.Y gRNA-*ADE2*.Y	[12]
pQC009	2µm *ampR KlURA3* gRNA-***XI-5***.Y	[13]
pQC030	2µm *ampR URA3* gRNA-***XI-1***.Y [2x]	[13]
pQC032	2µm *ampR URA3* gRNA-***XII-1***.Y [2x]	[13]
pQC033	2µm *ampR URA3* gRNA-***XII-5***.Y [2x]	[13]
pQC130	2µm *ampR URA3* gRNA-***XI-2***.Y [2x]	[13]
pQC133	2µm *ampR URA3* gRNA-***XII-3***.Y [2x]	[13]
pC10-15	2µm *ampR URA3* gRNA-***OPI1***.Y [2x]	[14]
pQC074	2µm *ampR URA3* gRNA-***X-2.***Y gRNA-***XII-2***.Y	This study
pQC197	2µm *ampR URA3* gRNA-***FAS1p***.Y [2x]	This study
pQC201	2µm *ampR URA3* gRNA-***PAH1***.Y [2x]	This study
pQC210	2µm *ampR URA3* gRNA-***HMX1***.Y [2x]	This study
pQC214	2µm *ampR URA3* gRNA-***ROX1***.Y [2x]	This study
pQC238	2µm *ampR KlURA3* gRNA-***ELP3***.Y	This study

Supplementary References

1. Keren, L. *et al.* Promoters maintain their relative activity levels under different growth conditions. *Mol Syst Biol* **9**, 701 (2013).
2. Katsuyama, Y., Miyahisa, I., Funa, N. & Horinouchi, S. One-pot synthesis of genistein from tyrosine by coincubation of genetically engineered *Escherichia coli* and *Saccharomyces cerevisiae* cells. *Appl Microbiol Biotechnol* **73**, 1143-1149 (2007).
3. Leonard, E. & Koffas, M.A. Engineering of artificial plant cytochrome P450 enzymes for synthesis of isoflavones by *Escherichia coli*. *Appl Environ Microbiol* **73**, 7246-7251 (2007).
4. Kim, D.H., Kim, B.G., Jung, N.R. & Ahn, J.H. Production of genistein from naringenin using *Escherichia coli* containing isoflavone synthase-cytochrome P450 reductase fusion protein. *J Microbiol Biotechnol* **19**, 1612-1616 (2009).
5. Kim, B.G. Biological synthesis of genistein in *Escherichia coli*. *J Microbiol Biotechnol* **30**, 770-776 (2019).
6. Trantas, E., Panopoulos, N. & Ververidis, F. Metabolic engineering of the complete pathway leading to heterologous biosynthesis of various flavonoids and stilbenoids in *Saccharomyces cerevisiae*. *Metab Eng* **11**, 355-366 (2009).
7. Chemler, J.A., Lim, C.G., Daiss, J.L. & Koffas, M.A. A versatile microbial system for biosynthesis of novel polyphenols with altered estrogen receptor binding activity. *Chem Biol* **17**, 392-401 (2010).
8. He, X.Z., Li, W.S., Blount, J.W. & Dixon, R.A. Regioselective synthesis of plant (iso)flavone glycosides in *Escherichia coli*. *Appl Microbiol Biotechnol* **80**, 253-260 (2008).
9. Koirala, N., Pandey, R.P., Thang, D.V., Jung, H.J. & Sohng, J.K. Glycosylation and subsequent malonylation of isoflavonoids in *E. coli*: strain development, production and insights into future metabolic perspectives. *J Ind Microbiol Biotechnol* **41**, 1647-1658 (2014).
10. Li, M.J. *et al. De novo* production of resveratrol from glucose or ethanol by engineered *Saccharomyces cerevisiae*. *Metabolic Engineering* **32**, 1-11 (2015).
11. Partow, S., Siewers, V., Bjorn, S., Nielsen, J. & Maury, J. Characterization of different promoters for designing a new expression vector in *Saccharomyces cerevisiae*. *Yeast* **27**, 955-964 (2010).
12. Mans, R. *et al.* CRISPR/Cas9: a molecular Swiss army knife for simultaneous introduction of multiple genetic modifications in *Saccharomyces cerevisiae*. *FEMS Yeast Res* **15** (2015).
13. Liu, Q. *et al.* Rewiring carbon metabolism in yeast for high level production of aromatic chemicals. *Nat Commun* **10**, 4976 (2019).
14. Liu, Y., Liu, Q., Krivoruchko, A., Khoomrung, S. & Nielsen, J. Engineering yeast phospholipid metabolism for *de novo* oleoylethanolamide production. *Nat Chem Biol* **16**, 197-205 (2020).
