# Supplementary material for: De novo biosynthesis of bioactive isoflavonoids by engineered yeast cell factories
Source: Nat Commun. 2021 Oct 19;12:6085. doi: 10.1038/s41467-021-26361-1 (PMC8526750; doi:10.1038/s41467-021-26361-1)
Supplement: Supplementary file 2 — Description of Additional Supplementary Files [file 41467_2021_26361_MOESM2_ESM.docx]

**Description of Additional Supplementary Files**

File Name: Supplementary Data 1
Description: *S. cerevisiae* strains used in this study.

File Name: Supplementary Data 2
Description: Codon-optimized genes used in this study.

File Name: Supplementary Data 3
Description: Primers used in this study.

File Name: Supplementary Data 4
Description: Assembled DNA constructs used in this study.
