# Supplementary material for: De novo biosynthesis of bioactive isoflavonoids by engineered yeast cell factories
Source: Nat Commun. 2021 Oct 19;12:6085. doi: 10.1038/s41467-021-26361-1 (PMC8526750; doi:10.1038/s41467-021-26361-1)
Supplement: Supplementary file 7 — Reporting Summary [file 41467_2021_26361_MOESM7_ESM.pdf]

nature portfolio

Corresponding author(s): Jens Nielsen
Last updated by author(s): Sep 23, 2021

Reporting Summary

Nature Portfolio wishes to improve the reproducibility of the work that we publish. This form provides structure for consistency and transparency in reporting. For further information on Nature Portfolio policies, see our Editorial Policies and the Editorial Policy Checklist.

Statistics

For all statistical analyses, confirm that the following items are present in the figure legend, table legend, main text, or Methods section.

n/a	Confirmed	
☐	☒	The exact sample size (n) for each experimental group/condition, given as a discrete number and unit of measurement
☐	☒	A statement on whether measurements were taken from distinct samples or whether the same sample was measured repeatedly
☐	☒	The statistical test(s) used AND whether they are one- or two-sided<br>*Only common tests should be described solely by name; describe more complex techniques in the Methods section.*
☒	☐	A description of all covariates tested
☒	☐	A description of any assumptions or corrections, such as tests of normality and adjustment for multiple comparisons
☐	☒	A full description of the statistical parameters including central tendency (e.g. means) or other basic estimates (e.g. regression coefficient) AND variation (e.g. standard deviation) or associated estimates of uncertainty (e.g. confidence intervals)
☐	☒	For null hypothesis testing, the test statistic (e.g. F, t, r) with confidence intervals, effect sizes, degrees of freedom and P value noted<br>*Give P values as exact values whenever suitable.*
☒	☐	For Bayesian analysis, information on the choice of priors and Markov chain Monte Carlo settings
☒	☐	For hierarchical and complex designs, identification of the appropriate level for tests and full reporting of outcomes
☒	☐	Estimates of effect sizes (e.g. Cohen's d, Pearson's r), indicating how they were calculated

Our web collection on statistics for biologists contains articles on many of the points above.

Software and code

Policy information about availability of computer code

Data collection	Chromeleon (version 6.80 DU10a Build 2826) was used for HPLC data collection and MS data was acquired with Agilent MassHunter LC-MS Data Acquisition (Version B.06.01).
Data analysis	Excel (Microsoft Office 365 Proplus) for quantitative analysis of HPLC data, CRISPRdirect webtool (http://crispr.dbcls.jp/) for gRNA design, and Agilent Masshunter Qualitative analysis (Version B.07.00 SP2) was used for MS data analysis.

For manuscripts utilizing custom algorithms or software that are central to the research but not yet described in published literature, software must be made available to editors and reviewers. We strongly encourage code deposition in a community repository (e.g. GitHub). See the Nature Portfolio guidelines for submitting code & software for further information.

Data

Policy information about availability of data

All manuscripts must include a data availability statement. This statement should provide the following information, where applicable:

- Accession codes, unique identifiers, or web links for publicly available datasets
- A description of any restrictions on data availability
- For clinical datasets or third party data, please ensure that the statement adheres to our policy

Data supporting the findings of this study are available within the article and its Supplementary Information files. The GenBank (https://www.ncbi.nlm.nih.gov/genbank/) accession numbers and codon-optimized nucleotide sequences of the genes referenced in this study are provided in this paper. Source data are provided with this paper as a Source Data file. All other data that support the findings of this study are available from the corresponding author upon request. All plasmids and strains used in this study are available from the corresponding author under a material transfer agreement.

Field-specific reporting

Please select the one below that is the best fit for your research. If you are not sure, read the appropriate sections before making your selection.

- [x] Life sciences
- [ ] Behavioural & social sciences
- [ ] Ecological, evolutionary & environmental sciences

For a reference copy of the document with all sections, see nature.com/documents/nr-reporting-summary-flat.pdf

Life sciences study design

All studies must disclose on these points even when the disclosure is negative.

Sample size	Biological triplicates were chosen as the standard for all experiments.
Data exclusions	No data was excluded.
Replication	Three biologically independent samples were performed for shake-flask fermentations on chemical production. All attempts at replication were successful.
Randomization	Not applicable. We performed metabolic engineering of a known yeast strain and used resultant strains for shake-flask cultivations, and thus randomization is not applicable.
Blinding	Not applicable. We rationally designed and carried out the experiments to achieve our goal and, therefore, blinding is not applicable in this study.

Reporting for specific materials, systems and methods

We require information from authors about some types of materials, experimental systems and methods used in many studies. Here, indicate whether each material, system or method listed is relevant to your study. If you are not sure if a list item applies to your research, read the appropriate section before selecting a response.

Materials & experimental systems

n/a	Involved in the study	
x		Antibodies
x		Eukaryotic cell lines
x		Palaeontology and archaeology
x		Animals and other organisms
x		Human research participants
x		Clinical data
x		Dual use research of concern

Methods

n/a	Involved in the study	
x		ChIP-seq
x		Flow cytometry
x		MRI-based neuroimaging
